# An IS*26*-assembled three-module chimeric plasmid co-localizing *bla*_NDM–1_ and *bla*_KPC–2_ undergoes post-transfer rearrangement in *Klebsiella pneumoniae*

**DOI:** 10.3389/fmicb.2026.1916718

**Published:** 2026-09-10

**Authors:** Zaikun Xiong, Rucai Chen, Caibing Zhao, Qianyi Cheng, Xianyao Liang, Ying Hu, Xu Yang, Jinyuan Yan, Wei He, Li Chen, Xinwei Huang, Pengfei Wang

**Affiliations:** 1 Department of Key Laboratory, The Second Affiliated Hospital of Kunming Medical University, Kunming, Yunnan, China; 2 Kunming Medical University Haiyuan College, Kunming, China; 3 Department of Laboratory Medicine, The Second Affiliated Hospital of Kunming Medical University, Kunming, Yunnan, China

**Keywords:** *bla*_KPC–2_, *bla*_NDM–1_, carbapenem-resistant *Klebsiella pneumoniae*, conjugative transfer, IS*26*-mediated cointegration, plasmid evolution, structural variation

## Abstract

**Introduction:**

Carbapenem-resistant *Klebsiella pneumoniae* (CRKP) poses a severe global public health threat, driven largely by plasmid-borne carbapenemase genes such as *bla*_NDM–1_ and *bla*_KPC–2_. Although CRKP strains co-producing multiple carbapenemases are increasingly reported, the co-localization of these genes on a single plasmid remains rare, and the evolutionary dynamics and stability of such hybrid resistance plasmids are poorly understood.

**Methods:**

In this study, we characterized a highly imipenem-resistant clinical ST11 CRKP isolate (W61) by combining whole-genome sequencing, conjugation assays, structural variation (SV) analysis, S1-PFGE, Southern blotting, and antibiotic susceptibility testing.

**Results:**

The isolate harbored a novel 224-kbp chimeric plasmid (pW61_1) and exhibited an imipenem minimum inhibitory concentration (MIC) of 512 μg/mL. Plasmid pW61_1 was composed of three modules fused through IS*26*-mediated cointegration: module 1 (IncFIB), module 2 (IncFII, *bla*_KPC–2_^+^), and module 3 (IncN2, *bla*_NDM–1_^+^). Under laboratory conditions, W61 maintained a high carbapenem-resistance retention rate in its native host even after 10 days of serial passage without antibiotic exposure. In addition, pW61_1 retained high structural stability after 10 days of serial passage under high imipenem concentrations. In the *K. pneumoniae* recipient, pW61_1 rearranged into smaller, resistance-retaining SVs, including SV-D3 (*bla*_NDM–1_^+^) and SV-D4 (*bla*_KPC–2_^+^), primarily through IS*26*-driven replication-associated excision. In the *Escherichia coli* J53 recipient, only small rearranged derivatives of pW61_1 were retained, each carrying one or both carbapenemase genes.

**Discussion:**

Our findings demonstrate that the tri-modular plasmid is transferable and undergoes post-transfer rearrangement under laboratory conditions. We hypothesize that this tri-modular plasmid may play an important role in the dissemination of carbapenemase genes, and therefore continued surveillance of this plasmid and its rearranged derivatives is warranted.

## Introduction

The global dissemination of CRKP poses a major threat to public health worldwide (Chen et al., 2024; Zhu et al., 2024). In CRKP, carbapenem resistance is primarily mediated by carbapenemases, notably the NDM and KPC enzymes (Aurilio et al., 2022). Carbapenemase-encoding genes, such as *bla*_NDM_ and *bla*_KPC_, are typically plasmid-borne and are frequently detected in *Enterobacterales* (Tilahun et al., 2021). Historically, such plasmids carried only a single carbapenemase gene (Wei et al., 2007; Fu et al., 2012). However, reports of CRKP isolates producing multiple carbapenemases have increased (Battikh et al., 2017; Protonotariou et al., 2018, 2019; Hu et al., 2022). In recent years, the isolation frequency of clinical CRKP co-harboring *bla*_NDM_ and *bla*_KPC_ has continued to rise (Zhang et al., 2025), although in most co-producing isolates the two genes reside on separate plasmids (Gao et al., 2020).

Antimicrobial resistance genes (ARGs) are closely associated with mobile genetic elements (MGEs), including plasmids, transposons, integrons, insertion sequences (ISs), integrative conjugative elements, and bacteriophages (Tang et al., 2024). MGEs promote ARG dissemination through diverse mechanisms, many of which are encoded by the elements themselves. MGEs can also co-localize multiple ARGs on a single genetic element through insertion, integration, or recombination, thereby giving rise to multidrug-resistant plasmids. However, the stability, replicative competence, and transferability of such plasmids remain poorly defined (Dionisio et al., 2019; Gao Y. et al., 2023). In particular, the contribution of MGEs to the dissemination of carbapenemase genes among *K. pneumoniae* isolates remains incompletely understood and warrants further investigation.

In this study, we characterized a CRKP isolate co-producing *bla*_NDM–1_ and *bla*_KPC–2_ that was recovered from the sputum of a 65-year-old female patient hospitalized in the neurosurgery department of a teaching hospital in Kunming, China. We performed comparative genomic analyses of a hybrid plasmid co-harboring *bla*_NDM–1_ and *bla*_KPC–2_ to elucidate its genetic context. We further evaluated the transferability of this plasmid and investigated the role of MGEs in reconfiguring its genetic composition in the donor versus recipient backgrounds.

## Materials and methods

### Clinical strains and antimicrobial susceptibility testing

**FIGURE 1:**
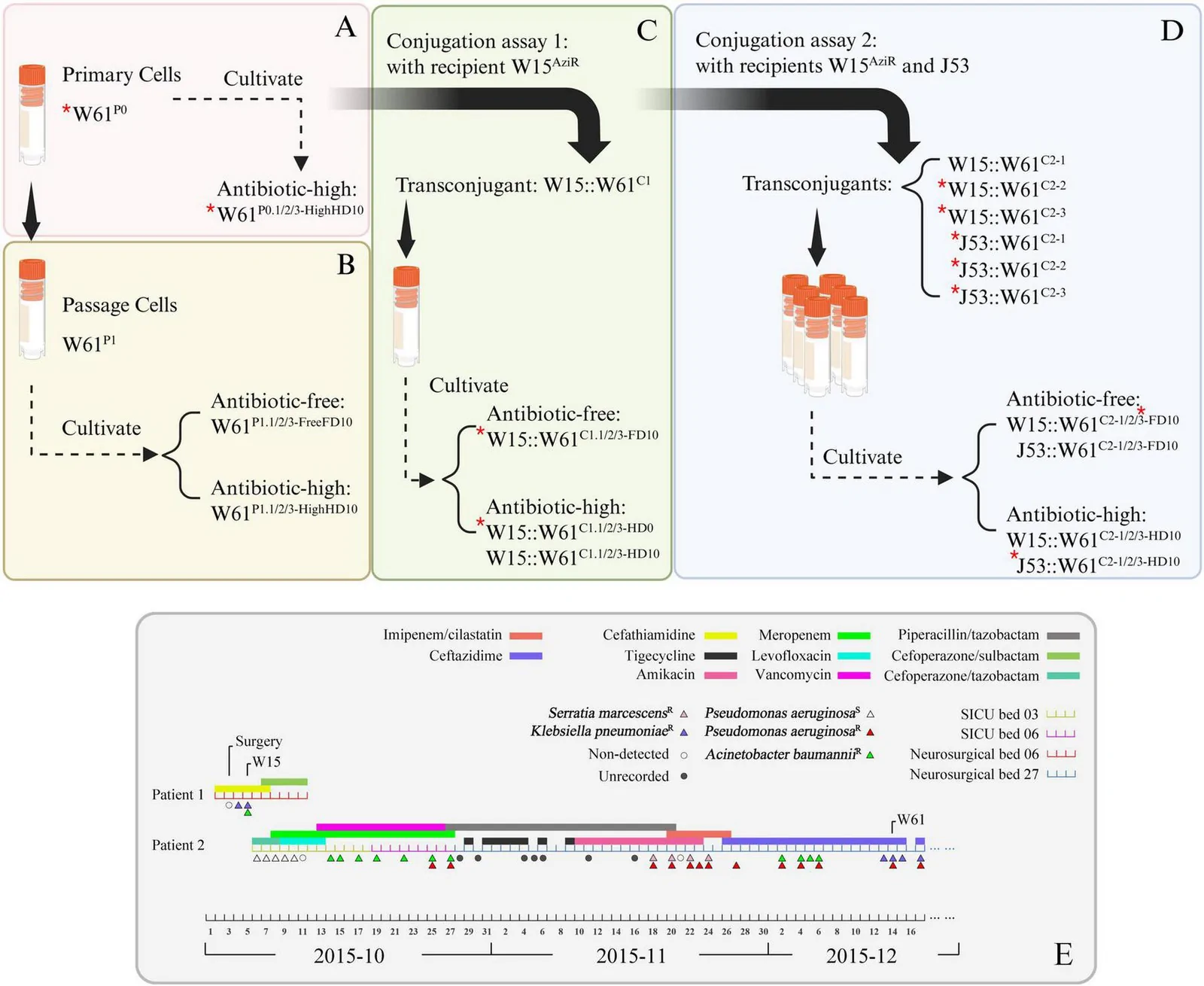
Sample-processing workflow and the patients’ clinical courses and medical treatment status. **(A)** Assessment of the antibiotic resistance plasmid stability in primary cells. **(B)** Evaluation of antibiotic resistance plasmid stability in P1 cells. **(C)** Analysis of plasmid alterations in the initial transconjugant. **(D)** Analysis of plasmid alterations among transconjugants from the second conjugation assay. The sample-labeling system is defined as follows: (1) C1, samples derived from the first conjugation assay; (2) C2, samples derived from the second conjugation assay; (3) C1.1, C1.2, and C1.3, three biological replicates of the C1 transconjugant; (4) C2-1, C2-2, and C2-3, three independent transconjugants isolated from the second conjugation assay; (5) -HD0, overnight culture under high-imipenem conditions; (6) -HD10, serial passage for 10 days under high-imipenem conditions; (7) -FD10, serial passage for 10 days under antibiotic-free conditions. Red asterisks indicate strains or strain groups subjected to Oxford Nanopore Technologies sequencing. **(E)** Sample collection information for W15 and W61. Triangles of different colors represent the time of detection of carbapenem-resistant bacteria. The separation times of the W15 and W61 samples are labeled above the timeline. Created in BioRender. Wang, P. (2026) https://BioRender.com/w6ltes9.

A 62-year-old male patient (patient 1) from Guizhou Province was admitted to the Emergency Department of the Second Affiliated Hospital of Kunming Medical University in 2015 due to coma and acute cerebral hemorrhage. Four hours after admission, the patient underwent bilateral external ventricular drainage via burr-hole trepanation and was subsequently transferred to the neurosurgery ward. On the first postoperative day, he developed mild pneumonia, and CRKP was detected in his sputum. The next day, his pneumonia worsened, and a carbapenem-susceptible strain (designated W15) was isolated from his sputum (Figure 1E). At the same period, a 65-year-old female patient (patient 2) was transferred to the SICU of the same hospital from another medical institution in the city due to severe traumatic brain injury and bilateral lung infection. She received combination antibiotic therapy throughout her hospitalization, which lasted several months. During the long-term recovery period after the patient was transferred to the neurosurgery ward, a CRKP strain W61 was isolated from her sputum sample (Figure 1E).

Both W61 and W15 were purified and archived in glycerol at −80 °C until 2024, when they were retrieved for sequencing and detailed analysis. Both strains were susceptible to sodium azide (MIC, 25 μg/mL), as determined by agar dilution. A substitution (Gly495Cys; GGC→TGT) was introduced into the *secA* gene of W15 by homologous recombination to generate a sodium azide-resistant mutant W15^AziR^ (Matsumura et al., 2018), which exhibited an MIC > 150 μg/mL (Supplementary method).

MICs of 14 antibiotics were determined for the donor, the recipient strains, and their corresponding transconjugants using the VITEK 2 Compact system and interpreted according to the 2023 Clinical and Laboratory Standards Institute (CLSI) guidelines, or U.S. Food and Drug Administration (FDA) breakpoints where CLSI criteria were unavailable (CLSI, 2023). For highly resistant strains, imipenem MICs were determined using the standard broth microdilution method. Briefly, imipenem was serially 2-fold diluted in fresh Mueller–Hinton broth in 96-well microtiter plates to yield final concentrations ranging from 0 to 1,024 μg/mL in a final volume of 100 μL. A single colony of each strain was grown overnight at 37 °C in LB broth supplemented with appropriate antibiotics, with shaking. Overnight cultures were diluted in fresh MH broth and adjusted to a 0.5 McFarland standard. The adjusted cultures were then diluted 1:100 to yield a final inoculum of 1 × 10^6^ CFU/mL, and 100 μL of each suspension was inoculated into wells. Three independent replicates were performed for each strain, with *K. pneumoniae* ATCC 10031 (plasmid-free and carbapenem-susceptible) serving as the control. Plates were incubated at 37 °C without shaking for 16–20 h, after which OD_600_ values were measured using a microplate reader (Thermo Multiskan Go). After background subtraction, the MIC was defined as the lowest imipenem concentration yielding an OD_600_ below 0.05.

### Carbapenem-resistance retention rate

Three independent W61^P0^ colonies were serially passaged for 10 days with a daily 1:1,000 dilution. Every 2 days, cultures were plated on antibiotic-free LB agar or LB agar containing 4 μg/mL imipenem to quantify carbapenem-resistance retention. Two clinical CRKP strains, KP21 carrying *bla*_KPC–2_-positive p21_1 and KP82 carrying *bla*_NDM–1_-positive p82_4, were used as controls. The carbapenem-resistance retention rate was calculated as the percentage of CFU recovered on imipenem-containing agar relative to that recovered on antibiotic-free agar. Three replicates were conducted for each strain, and data are presented as means ± standard deviation (SD) (*n* = 3).

### Plasmid structural stability and conjugation assays

To assess the structural stability of the hybrid plasmid in its native host, one W61^P0^ colony was cultured overnight in LB broth containing 4 μg/mL imipenem. The overnight culture was divided into three aliquots, each of which was passaged for 10 days using daily 1:1,000 dilution under high-imipenem conditions (Figure 1A). Because the transconjugants used in subsequent experiments exhibited different levels of imipenem resistance, we applied a strain-specific ½ × MIC of imipenem rather than a single fixed concentration to maintain sustained high antibiotic pressure throughout the passaging process (the concentrations used are listed in Supplementary Table 6).

Because the volume of cryopreserved W61^P0^ cells was limited, one W61^P0^ colony was expanded and preserved as P1 cells (W61^P1^). As described for W61^P0^, the overnight culture of W61^P1^ was divided into six aliquots. Three aliquots were passaged under high-imipenem conditions, and the remaining three were passaged under antibiotic-free conditions (Figure 1B).

Two independent conjugation assays were performed using the filter-mating method with minor modification (Figures 1C, D; Al-Muzahmi et al., 2023). In brief, after strain recovery and colony selection, recipient strains W15^AziR^ and J53 and donor strain W61^P0^ were separately inoculated into LB broth containing 150 μg/mL sodium azide or 4 μg/mL imipenem, respectively, and cultured overnight at 37 °C with shaking until reaching a 1.0 McFarland standard. Subsequently, 1 mL of each culture was centrifuged at 10,000 × *g* for 2 min to harvest the cells. The pellet was washed twice with 1 mL of 1 × Phosphate Buffered Saline (PBS) and resuspended in 1 mL of 1 × PBS. A sterile 0.45 μm pore-size membrane was placed on an antibiotic-free LB plate. Aliquots (100 μL) of the recipient, donor, and their mixed bacterial suspensions were spotted onto the center of the filter. The plates were allowed to dry and then incubated overnight at 37 °C. Subsequently, the filter was removed from each LB plate, placed into a sterile 50 mL tube, and washed with 1 mL of 1 × PBS to detach the cells. Each cell suspension was serially diluted up to 10^8^ in 1 × PBS. Transconjugants were selected on LB agar containing 150 μg/mL sodium azide and 4 μg/mL imipenem and were verified by Polymerase Chain Reaction (PCR) for the presence of *bla*_NDM_ and *bla*_KPC_. Dilutions of the donor were plated on LB with 4 μg/mL imipenem and the recipient with 150 μg/mL sodium azide. Transfer frequency was calculated as the number of transconjugant CFU divided by the number of recipient CFU. Three replicates were conducted for each recipient, and the data are presented as means ± standard deviation (SD) (*n* = 3).

### High-throughput sequencing

To analyze the structural variations of the hybrid plasmid among the populations of the original host, transconjugants, and serially passaged transconjugants under different antibiotic conditions, we performed high-throughput sequencing of selected cultures, indicated by red asterisks in Figure 1. Genomic DNA was extracted using the TIANamp Bacterial DNA Kit (Tiangen). DNA was quantified using a fluorescent dye assay (Quant-iT PicoGreen dsDNA Assay Kit), and its integrity was assessed by 1% agarose gel electrophoresis. Long-read sequencing libraries were generated using the SQK-LSK109 ligation kit (Oxford Nanopore Technologies, UK). For error correction, Illumina NovaSeq libraries (TruSeq DNA Sample Preparation Kit, Illumina, USA) with 400 bp inserts and Paired-end 2 × 150-bp reads were sequenced to ≥100 × coverage. Sequencing was performed by Personal Biotechnology Company (Shanghai, China) using the Nanopore PromethION 48 and Illumina NovaSeq platforms, respectively. Raw PE sequencing data were processed with Adapter Removal (V2.2.2) for adapter contamination removal and data filtering (–max-ns *n* = 5 –min-length length = 50 –min-mean-quality 20) (Schubert et al., 2016). Flye (v2.9.1-b1781) (Kolmogorov et al., 2019) and Unicycler (v0.5.0) (Wick et al., 2017) software were used to assemble the Nanopore sequencing reads. Using high-quality filtered paired-end data, the Nanopore assemblies were polished with pilon (v1.22) (Walker et al., 2014) to refine the contigs and generate the complete sequence.

### Bioinformatics analysis

Multilocus sequence typing (MLST) and capsular (*wzi*) typing were performed using the *Klebsiella* BIGSdb database^1^ (Brisse et al., 2013). Virulence factors and resistance-associated genes were annotated by aligning gene sequences against the Virulence Factor Database (VFDB^2^) (Zhou et al., 2025) and the Comprehensive Antibiotic Resistance Database (CARD^3^) (Alcock et al., 2020) using BLAST (v2.17.0+). Plasmid sequences were compared against the NCBI plasmid reference database^4^ using BLAST in CLC Genomics Workbench v23.0.4. Based on the coverage of each match in BLAST Graphics, relevant plasmids were retrieved by accession number, uniformly oriented, annotated using RAST 2.0, and visualized with Easyfig (v2.2.5). Plasmid maps were generated using Proksee^5^ with annotations from CARD, mobileOG-db, and Prokka tools and were cross-verified by NCBI BLASTp. Insertion sequences were identified using ISFinder. ^6^

Structural variation (SV) detection was performed for all ONT data to identify plasmids with novel structures. Among them, four datasets were used to identify predominant SV types: DS-W61^P0^, which assessed SVs in primary cells; DS-W61^P0.1/2/3–HD10^, which assessed changes in primary cells after serial passage under high-imipenem conditions; DS-W15::W61^C1.1/2/3–FD10^, which assessed changes in the first transconjugant after serial passage under antibiotic-free conditions; and DS-W15::W61^C1.1/2/3–HD0^, which assessed changes in the first transconjugant after overnight culture under high-imipenem conditions. ONT reads from each sample were trimmed in CLC Genomics Workbench v23.0.4 using a quality-score limit of 0.12, discarding reads < 2,000 bp. Trimmed reads were mapped to the corresponding reference genomes using minimap2 and converted into sorted and indexed BAM files with SAMtools. SVs were called using sniffles (v1.0.12) with the following parameters: –skip_parameter_estimation -l 200 -s 3 -n -1 -v (to avoid filtering out some rare but important SV types, we set the minimum read-support threshold to 3). For each SV ID, supporting reads were extracted using seqtk to generate SV-specific FASTQ files, which were remapped to its reference sequence for visualization and manual validation in IGV (v2.16.2) and NCBI BLAST tool.^7^ The main aspects requiring to manual check include: (1). completeness of sequence alignment (whether reads are clipped and whether clipping results from recombination or poor sequence quality at read ends); (2). sequence alignment similarity should be greater than 90% (excluding contaminations); (3). consistency of SV boundaries (redefining boundaries affected by sequencing errors or alignment errors); (4). excluding regions with high-frequency genomic variations (e.g., phage-related regions); (5). excluding false positives reported by the software. When necessary, CLC Genomics Workbench was used to remap ONT reads to pW61_1 reference sequence to examine read coverage.

### PCR identification, S1-PFGE, and Southern blotting

PCR experiments were performed to validate identified SVs and identify the potential circularized intermediates in different treatment groups. Primer sequences are listed in Supplementary Table 1. To evaluate the evolutionary dynamics of SV-D2 in the W61^P1.1/2/3−HD10^ and W61^P1.1/2/3−FD10^ groups, specific primers were used to detect the tandem repeat status of the *bla*_KPC–2_ gene cluster (primers 1F/1R amplified the 6,707 bp single-copy *bla*_KPC–2_ cassette and the 11,453 bp two-copy *bla*_KPC–2_ cassette, while primers 2F/2R detected both the two-copy *bla*_KPC–2_ cassette and the circularized single-copy *bla*_KPC–2_ cassette (Supplementary Figure 1A). PCR experiments was performed using *Takara LA Taq* Polymerase (TaKaRa, Japan) under the conditions of 94 °C for 2 min, 35 × (94 °C for 30 s, 60 °C for 30 s and 72 °C for 3–12 min according to the amplicon size), and 72 °C for 5 min. All DNA templates were normalized to an equal concentration (10 ng/μL), and the densities of the amplified bands were assessed by visual inspection. S1-PFGE and Southern blotting were used to verify circularized SVs and additional plasmids, as described previously (Hu et al., 2022).

### Statistical test

Unpaired *t*-test was employed to analyze the differences in retention rates between different samples at each single time point.

## Results

### Genetic features of W61 and W15

Strain W61 harbored four plasmids. Among these, pW61_1 carried IncFIB (pB171), IncFII (pHN7A8), and IncN2 replicons, confirming its multireplicon and hybrid nature. A total of 75 virulence-associated genes and 72 antimicrobial-resistance/efflux-related genes were located on the chromosome. No virulence-associated genes were detected in extra chromosomal elements. Plasmid pW61_1 encoded 11 ARGs, including *bla*_NDM–1_ and *bla*_KPC–2_, whereas the other three plasmids encoded no ARGs. Strain W15 also carried four plasmids, among which pW15_1 encoded four ARGs and harbored IncFIB(K)/IncFII(K) replicons commonly found in *K. pneumoniae* (Navon-Venezia et al., 2017; Wyres et al., 2020). Genomic features, VFDB results, and CARD results for the two strains are summarized in Supplementary Table 2. Both strains belonged to ST11 and capsular type K14.K64 (wzi64). Their chromosomal sequences were highly similar (Supplementary Figure 2), differing by only 11 single-nucleotide polymorphisms (SNP) and four IS-associated small-scale structural variations (Supplementary Table 3). Their plasmid profiles were also highly similar, with the exception of pW15_1 and pW61_1 (Supplementary Figure 2).

### Genetic and comparative analyses of hybrid plasmid pW61_1 co-harboring *bla*_NDM–1_ and *bla*_KPC–2_

**FIGURE 2:**
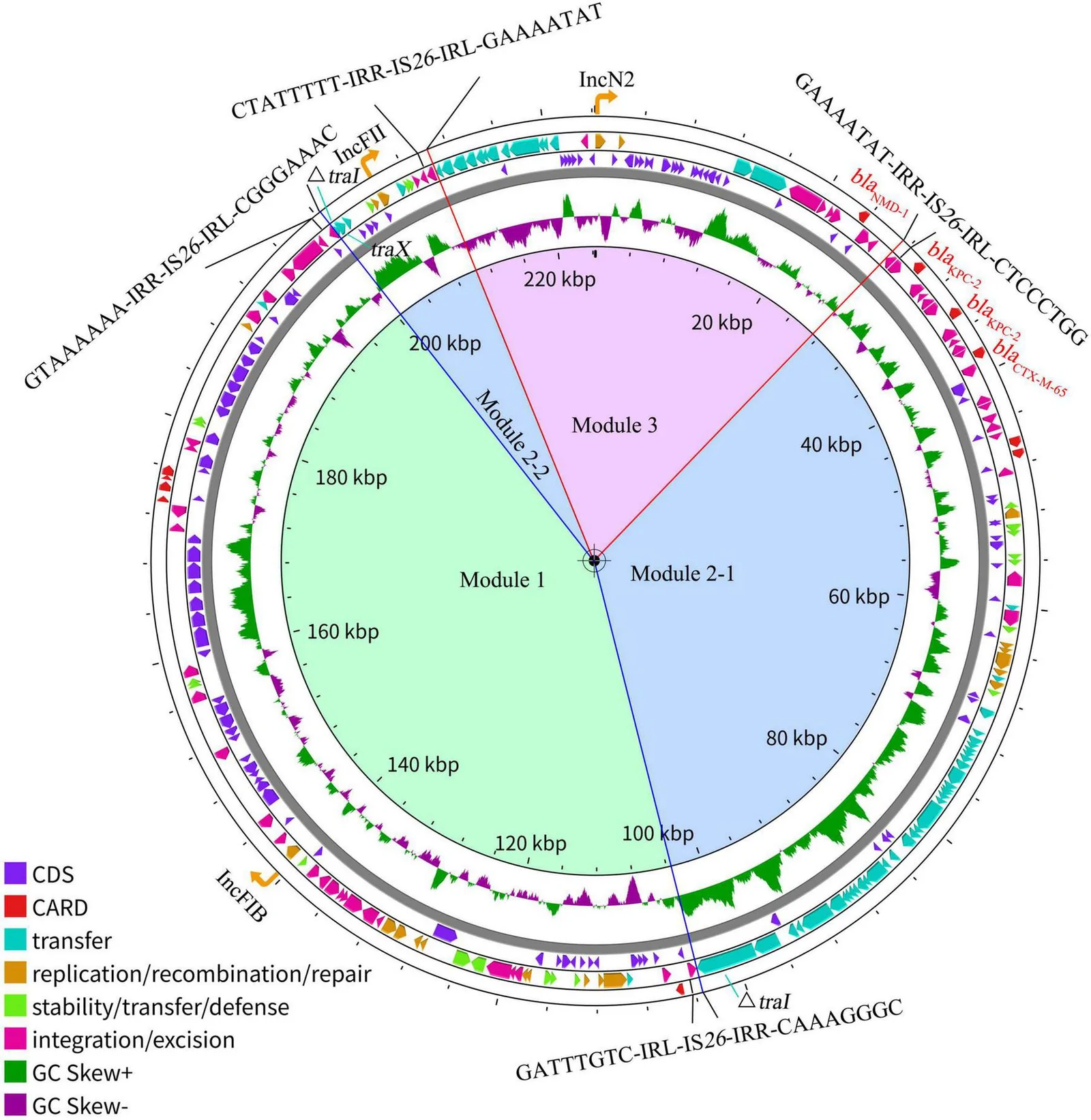
Genetic structure of the *bla*_NDM–1_ and *bla*_KPC–2_ co-carrying three-module chimeric plasmid pW61_1. The range of each module is indicated by the module name and corresponding region on the circular plasmid map. IS*26* elements located at module boundaries are highlighted, with their IRs and corresponding DRs annotated in the 5′ to 3′ direction.

**FIGURE 3:**
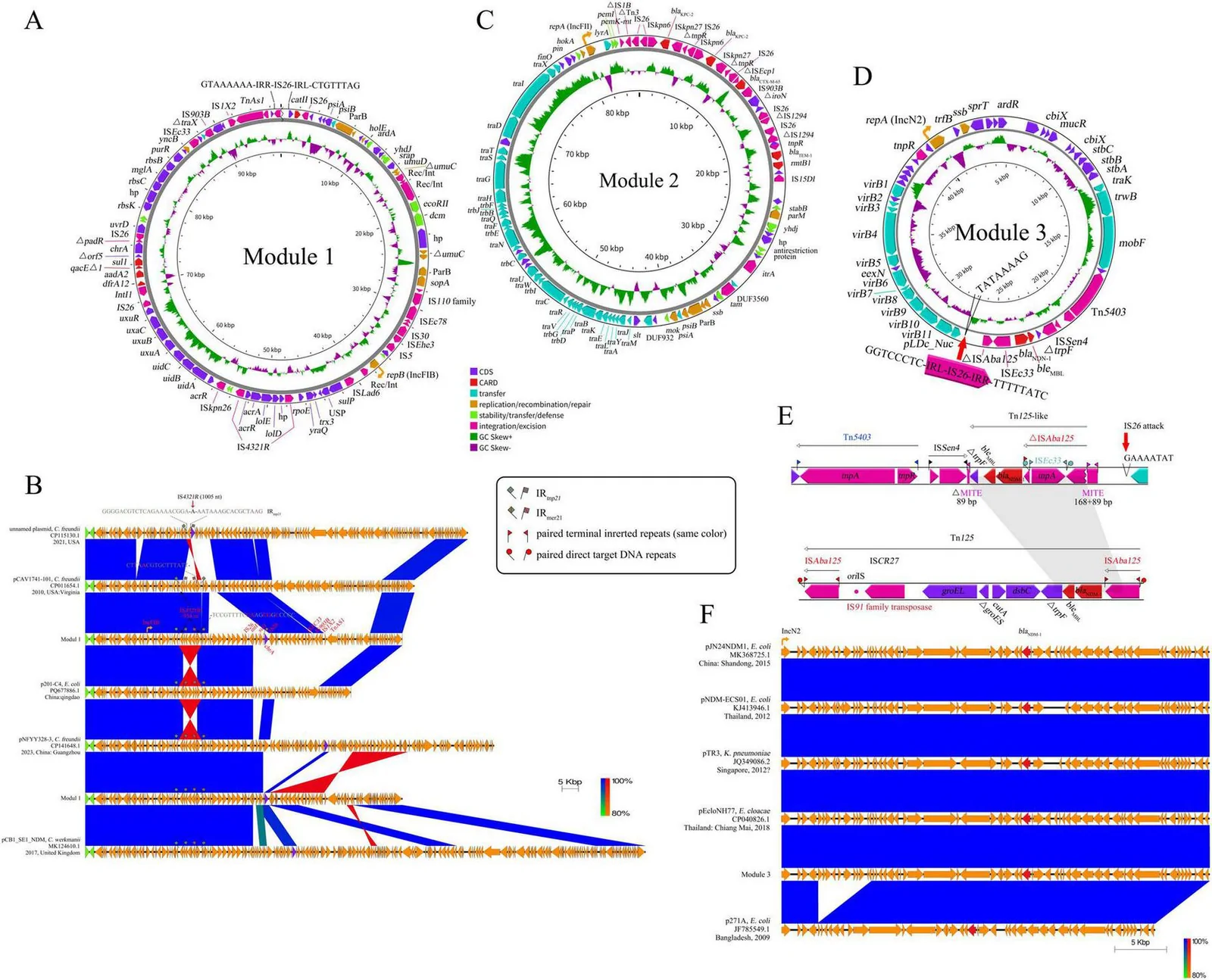
Genetic analysis and homology comparison of each module. **(A)** The genetic features of the predicted circular module 1. The IS*26* located at the closed boundary of module 1 is marked according to its 5′ to 3′ orientation. **(B)** Comparative analysis of module 1 with representative structurally similar plasmids. **(C)** Genetic features of predicted circular module 2. **(D)** Genetic features of the predicted circular module 3. The IS*26* targeted site is marked with a red arrow. **(E)** Comparative analysis illustrating the origin of the *bla*_NDM–1_ harboring Tn*125*-like element. **(F)** Comparative analysis of module 3 with representative structurally similar plasmids.

Comparative analysis revealed that pW61_1 consisted of three major functional modules (Figure 2), each showing high similarity to several known plasmids in public databases. Module 1 contained an IncFIB (pB171) replicon (pMLST allele = 70) and showed >99% sequence identity to numerous plasmids but with only limited query coverage (40%–60%) (Figures 3A, B). Among the matched plasmids, those from *Citrobacter freundii* were the most frequently represented (Supplementary Table 4).

**FIGURE 4:**
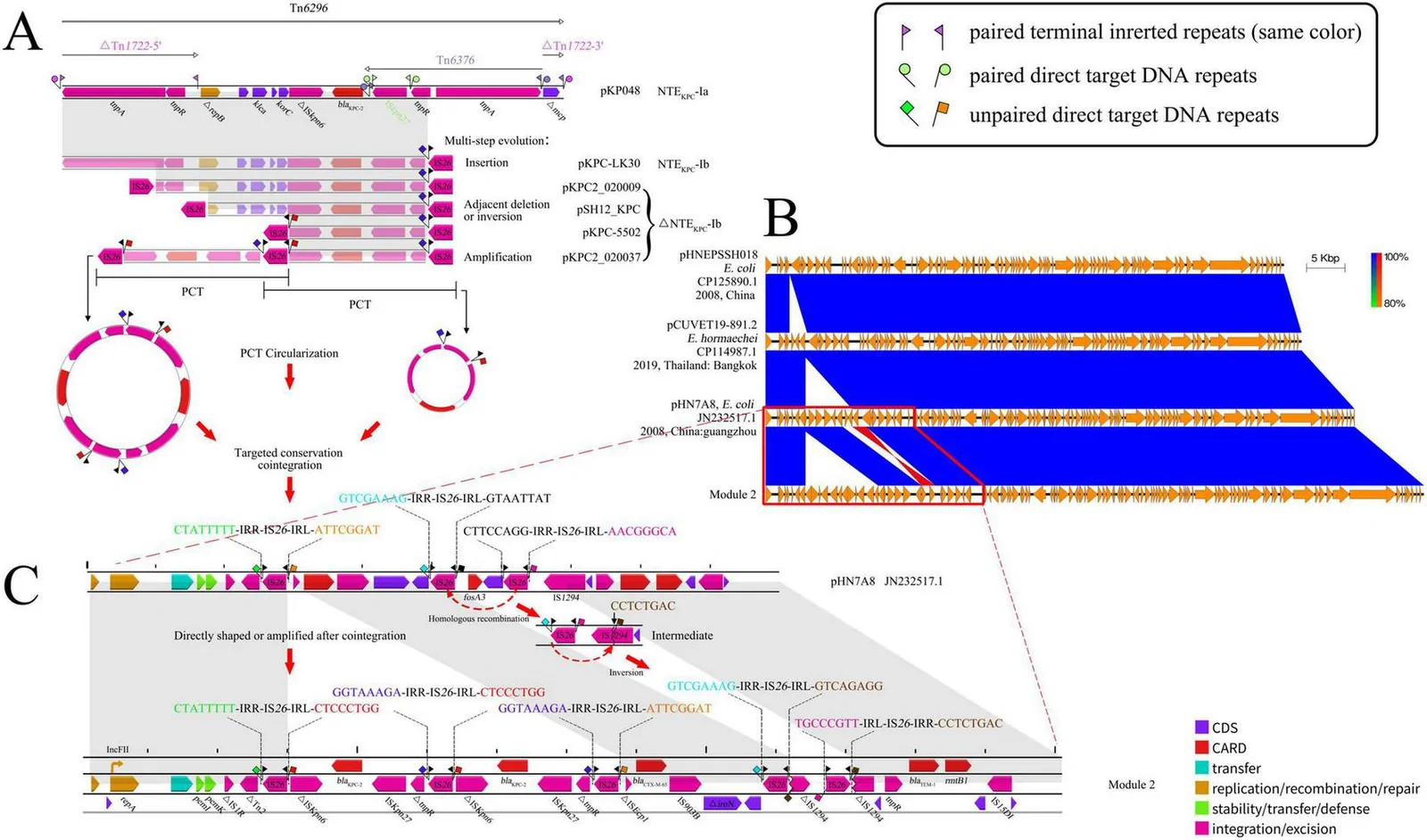
IS*26*-mediated formation and duplication of the *bla*_KPC–2_-harboring cassette and loss of *fosA3* in module 2. **(A)** Schematic diagram illustrating the origin of the *bla*_KPC–2_-harboring cassette in module 2. **(B)** Comparative analysis of pHN7A8-like plasmids. **(C)** Comparative analysis illustrating the formation and duplication of the *bla*_KPC–2_-harboring cassette and loss of *fosA3* in module 2.

Module 2 carried an IncFII (pHN7A8) replicon (pMLST allele = 33) and encoded several IS*26*-associated ARGs, including *bla*_KPC–2_, *bla*_TEM–1_, *bla*_CTX–M–65_, and *rmtB1*, as well as multiple conjugative-associated genes (Figures 2, 3C). Module 2 showed high sequence identity to pHN7A8, originally identified in an *E. coli* strain isolated from a dog in China in 2008 (Figure 4B; He et al., 2013). It also showed high coverage and identity (99%–100%) to specific regions of larger plasmids (140–154 kbp) (Li et al., 2022; Xia et al., 2022; Han et al., 2024). These findings suggest that module 2-like elements can replicate autonomously and frequently integrate into other plasmids.

Module 3 contained stability genes (*stbA*, *stbB*, and *stbC*), conjugative-associated genes, a type IV secretion system (T4SS; *virB1*-*virB11*), and a *bla*_NDM–1_-carrying non-classical Tn*125* derivative (Figures 2, 3D, E). This module exhibited 99.99% sequence identity to known IncN2 plasmids prevalent in the Asia-Pacific region (Chen et al., 2012; Netikul et al., 2014; Khong et al., 2016; Hao et al., 2019; Figure 3F), suggesting that module 3-like plasmids can disseminate widely following acquisition of *bla*_NDM–1_ (Acman et al., 2022; Cai et al., 2026).

### Genetic analyses of key MGEs in hybrid plasmid pW61_1

**FIGURE 5:**
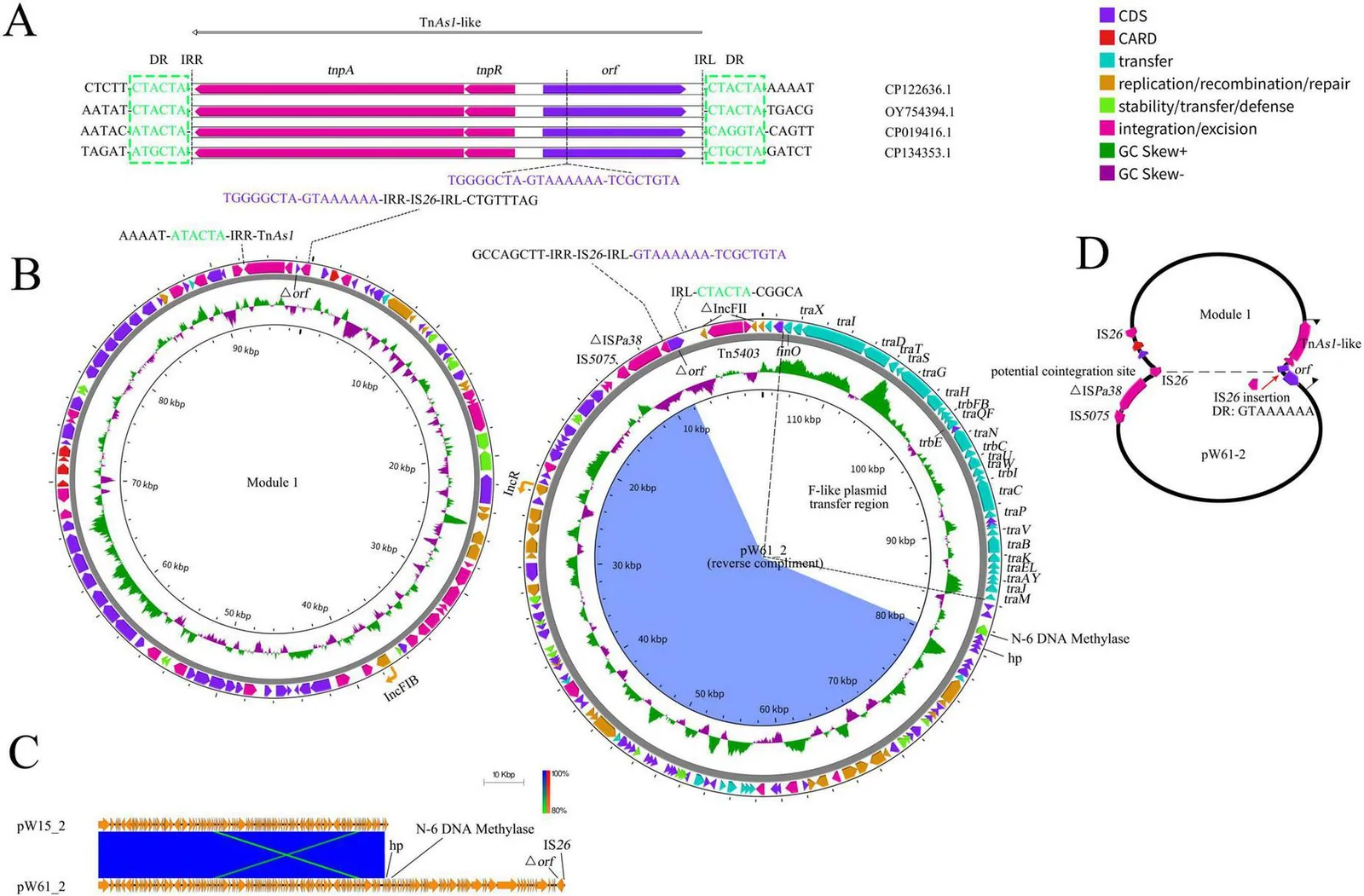
IS*26*-mediated formation of plasmid pW61_2. **(A)** Comparative analysis of several published plasmids with similar Tn*As1*-like transposons. **(B)** The integration boundaries between module 1 and pW61_2. **(C)** Comparative analysis of pW15_2 and pW61_2. **(D)** Schematic diagram of the original composite plasmid formed by module 1 and pW61_2. The formation of plasmid pW61_2 is proposed to involve the following steps: (1) acquisition of a module 1-related segment by a pW15_2-like plasmid; (2) insertion of an IS*26* into the passenger gene of the Tn*As1*-like transposon, generating an 8-bp DR; (3) formation of a mobile TU through homologous recombination between two IS*26* elements, facilitating module 1 excision and pW61_2 generation. The green dashed box and green font indicate the partially conserved 6-bp DRs at the target site. The purple font within the *orf* gene highlights the recombination site.

Module 1 contained a truncated Tn3 family transposon (Tn*As1*-like), whose transposase and resolvase genes were highly similar to these of canonical Tn*As1* recorded in the ISfinder database, although its passenger gene (orf) differed (Figure 5B, left). This TnAs1-like element appeared to be capable of generating partially conserved 6-bp target-site direct repeats (DRs) (Figure 5A). However, in the present study, the element lacked the region spanning from the 3’ terminus of its passenger gene (orf) to its left inverted repeat (IRL), owing to an IS*26* integration (Figure 5B, left). Notably, the missing sequence matched the region upstream of an IS*26* in plasmid pW61_2 from the same host (Figure 5B, right). Furthermore, the two IS*26* elements shared an identical 8-bp DR: GTAAAAAA, suggesting the prior existence of a module 1-pW61_2 complex (Figure 5D). Homology comparison of pW61_2 and pW15_2 identified a highly conserved region (Figure 5C), while one end of the divergent region aligned precisely with the Tn*As1*-like transposon disruption site.

Boundary analysis between module 1 and module 2 identified a truncated *traI* with two IS*26* elements in the same orientation and sharing an identical 8-bp DR: CGGGAAAC (Figure 2). These findings indicate that the fusion of module 1 and module 2 is consistent with IS*26*-mediated cointegration model: module 1 was likely excised first from the ancestral module 1-pW61_2 complex and subsequently integrated into module 2.

In module 2, we identified a tandem repeat region carrying two copies of the *bla*_KPC–2_ cassette (Figure 4C), with an 8-bp DR: CTATTTTT at the 5′ end and a second 8-bp DR: ATTCGGAT at the opposite end. In pHN7A8, the same two DRs flanked an IS*26* at a position identical to that in module 2, whereas the tandem repeat region was absent (Figure 4C). Based on the genetic features surrounding the *bla*_KPC–2_ gene, this tandem repeat region belonged to a △NTE_KPC_-Ib transposon (Figure 4A; Chen L. et al., 2014; Chen Y.-T. et al., 2014). Homology analysis indicated that this configuration likely originated from a Tn*6296* derivative that evolved through multiple steps and is hypothesized to have been inserted into the pHN7A8 backbone via IS*26*-mediated targeted conservative cointegration (Figure 4A). Module 2 also exhibited deletion of *fosA3* accompanied by inversion of an adjacent IS*26*, whereas in pHN7A8 *fosA3* was flanked by two IS*26* elements in direct orientation. This rearrangement can be explained by IS*26*-mediated homologous recombination, which first deleted *fosA3*, followed by an inversion event, ultimately resulting in an 8-bp DR: CCTCTGAC and a truncated IS*1294* (Figure 4C).

Module 3 contained a *bla*_NDM–1_ gene within an integration region organized as Tn*5403*-IS*Sen4*-△MITE*-*△*trpF*-*ble*_MBL_-*bla*_NDM–1_-△IS*Aba125*/IS*Ec33*-MITE, containing a remnant of MITE-mediated Tn*125*-like element (Figure 3E), which has been well-characterized in previous studies (Hu et al., 2012; Partridge and Iredell, 2012; Sun et al., 2015). This module was flanked by two intact IS*26* elements in the same orientation with identical terminal 8-bp DRs: GAAAATAT (Figure 2). Sequence analysis revealed that module 3 was highly similar to its homologous plasmid, suggesting its potential for independent replication (Figure 3F). More importantly, none of these plasmids were found to contain IS*26*. We therefore hypothesized that module 3 may be targeted by module 2, or by the module 1-module 2 complex, through IS*26*-mediated copy-in cointegration (Figures 3D, F).

### Carbapenem-resistance retention rate

During the 10-day passage in antibiotic-free medium, the ratio of CFU grown on imipenem-containing LB agar to those grown on antibiotic-free LB agar remained similar among the three tested strains across the first five time points, with no significant differences, indicating negligible plasmid loss (Supplementary Figure 3). For W61, the carbapenem-resistance retention rate declined to 82.33 ± 2.15% by day 10, a significant decrease relative to the other two strains.

### Plasmid structural stability and antibiotic susceptibility

**FIGURE 6:**
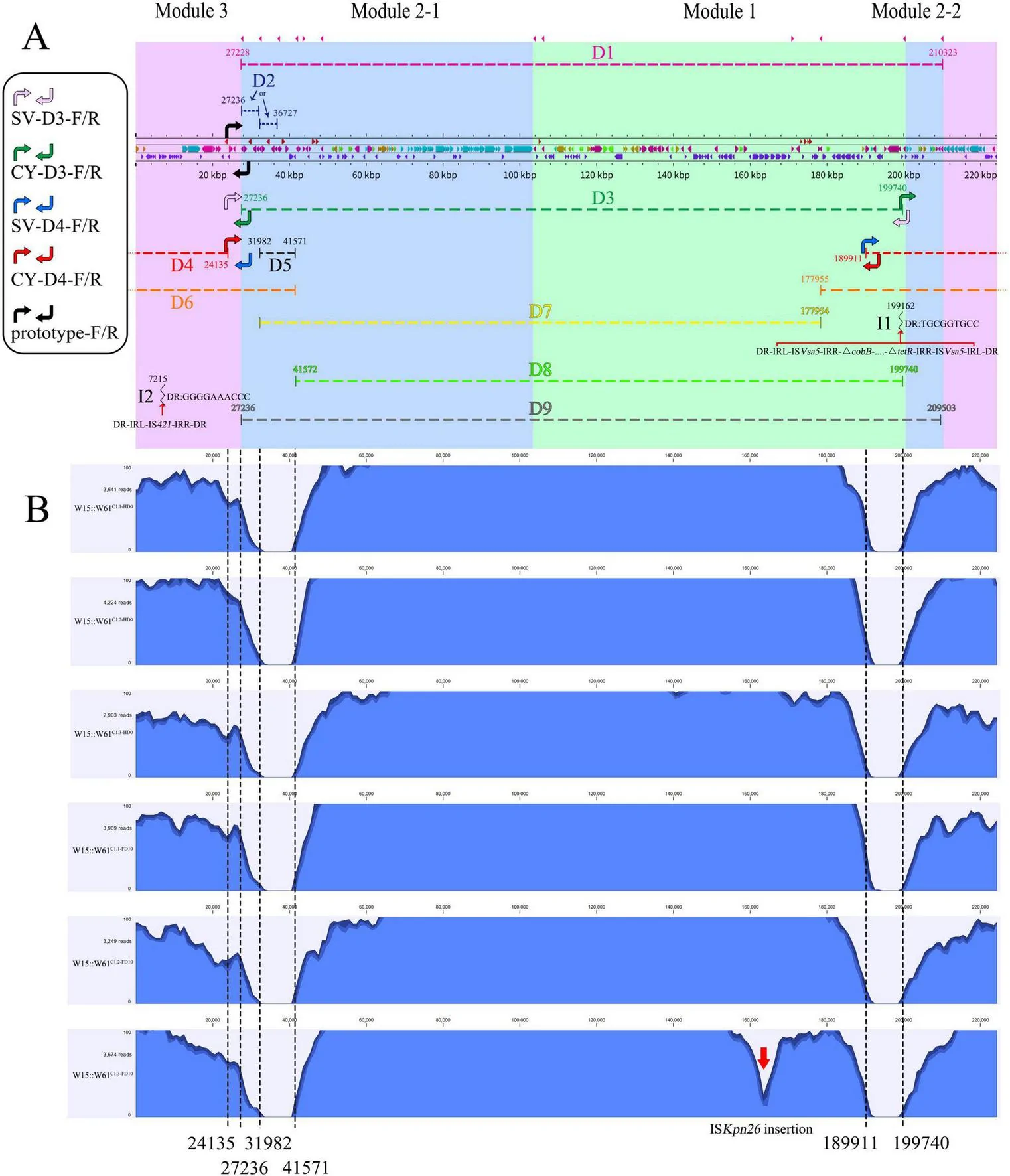
Structural variations identified across different transconjugants and the validation of the structural non-complementarity of SV-D3 and SV-D4 by ONT read mapping. **(A)** The schematic diagram of SV genetic structures. IS*26* elements are highlighted at the top with purple triangles. Dashed lines indicate regions absent from the corresponding SV. Excisions in some SVs may have alternative boundaries because DRs and insertion elements at the boundaries can align to either end. SV-D5 did not represent an independent mutation class, as only three reads spanning the *bla*_NDM–1_ and *bla*_KPC–2_ region were detected across datasets, suggesting early loss of the D5 segment during transconjugant formation. Most SV-D5 structures were associated with SV-D4. **(B)** ONT read mapping for DS-W15::W61^C1.1/2/3−HD0^ and DS-W15::W61^C1.1/2/3−FD10^.

**FIGURE 7:**
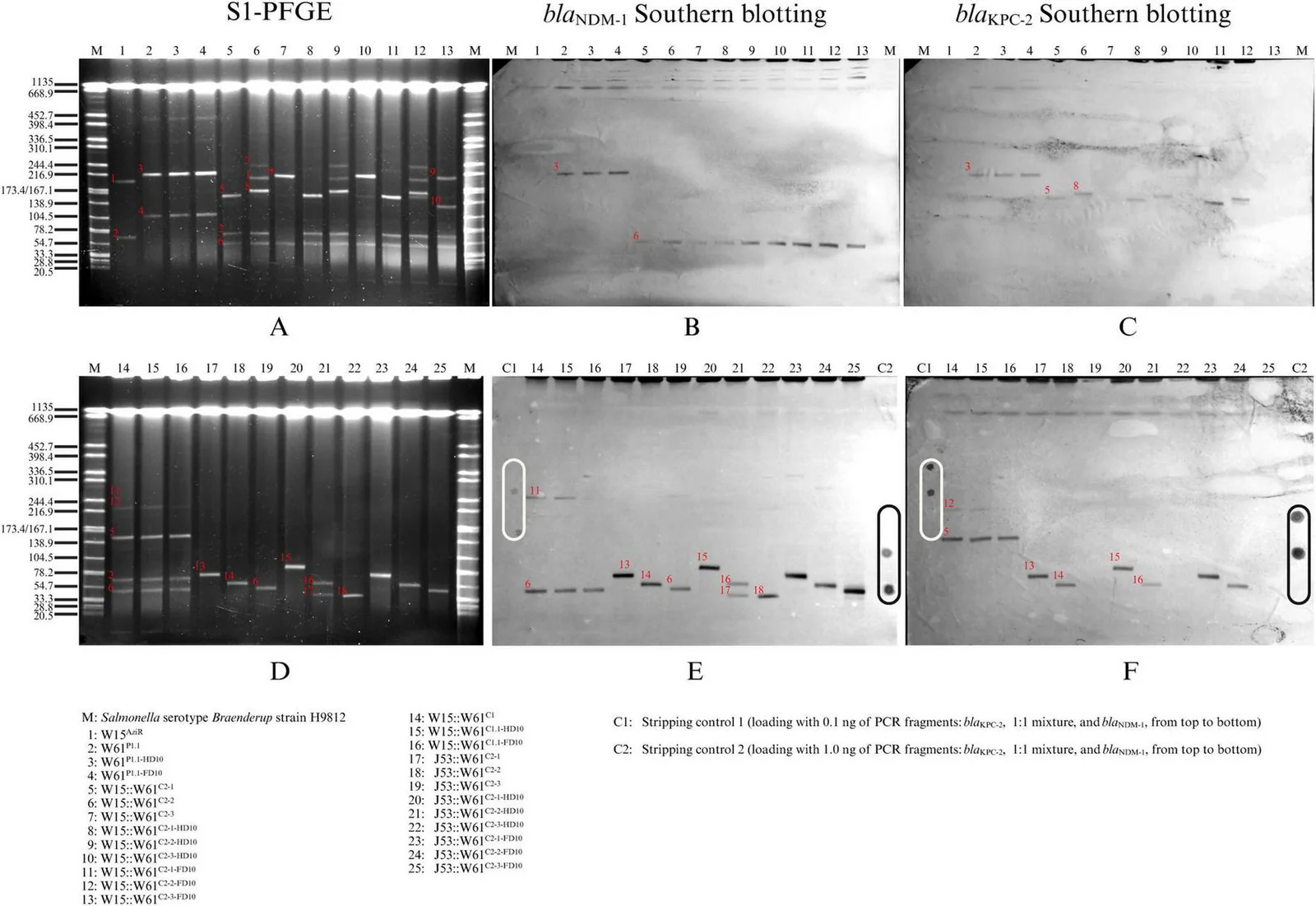
S1-PFGE profile and *bla*_NDM–1_/*bla*_KPC–2_ Southern blotting. **(A,D)** S1-PFGE profiles of the corresponding strains. **(B,E)** First-round *bla*_NDM–1_ Southern blotting results. **(C,F)** Second-round *bla*_KPC–2_ blotting results after membrane regeneration. The sizes and characteristics of each identified band are summarized in Supplementary Table 6. Created in BioRender. Wang, P. (2026) https://BioRender.com/1wiwvu8.

Although SV detection identified two primary variants in W61^P0^, both were supported by only a small number of reads (Supplementary Table 5). SV-D1 was supported by only three reads, substantially fewer than SV-D2. SV-D1 represented a rare plasmid-like module 3 structure that may correspond to a residual, unintegrated plasmid, as it lacked IS*26* (Figure 6A). SV-D2 (219,540 bp) lacked one of the two D2 segments (i.e., the tandem repeat region of the *bla*_KPC–2_ cassette). In the DS-W61^P0.1/2/3–HD10^, only SV-D2 was consistently detected, indicating a shift toward a single-copy *bla*_KPC–2_ cassette (Supplementary Table 5). However, among the three independent lineages, one yielded results similar to those observed in primary cells (i.e., a limited number of SV-D2 supporting reads) (Supplementary Figure 4). PCR was used to confirm the presence of single-copy and two-copy *bla*_KPC–2_ cassette in the four samples. The results showed that the single-copy *bla*_KPC–2_ cassette was stably detectable in all samples, while the two-copy *bla*_KPC–2_ cassette was only detected in the W61^P0^ and W61^P0.3–HD10^. These results were consistent with SV detection and read mapping (Supplementary Figure 1B). In S1-PFGE analysis, band 3 in W61^P1.1^ and its derivatives corresponded to SV-D2, whereas band 4 corresponded to pW61_2; no other variants were detectable (lanes 2–4 in Figures 7A–C). These results indicate that pW61_1 maintained high structural stability in its native host, with SV-D2 becoming predominant under high antibiotic pressure.

Conjugation assays confirmed the transferability of pW61_1, with transfer frequencies of (1.10 ± 0.29) × 10^–4^ to W15^AziR^ and (4.86 ± 2.06) × 10^–4^ to *E. coli* J53. Antibiotic susceptibility testing showed that both W61^P0^ and W61^P1^ were resistant to all tested antibiotics, with imipenem MICs of 512 μg/mL, but remained susceptible to tigecycline. The two strains exhibited identical resistance profiles. As expected, W15 and W15^AziR^ were susceptible to most agents, whereas all transconjugants exhibited variably increased imipenem resistance (Table 1).

**TABLE 1:** Resistance profiles.

Strains	AMK	AMC	FEP	CSL	FOX	CAZ	CRO	CXM	ETP	IMP	TZP	LVX	TGC	SXT
W15	<2	8	2	<8	>64	1	1	32	0.25	<0.25	16^#^	>8	2	>320
W15^AziR^	<2	4	2	<8	>64	0.5	1	16*	0.25	<0.25	16^#^	>8	2	>320
W61^P0^	>64	>32	>32	>64	>64	>64	>64	>64	>8	512	>128	>8	2	>320
W61^P1^	>64	>32	>32	>64	>64	>64	>64	>64	>8	512	>128	>8	2	>320
W15::W61^C1^	>64	>32	>32	>64	>64	>64	>64	>64	>8	256	>128	>8	2	>320
W15::W61^C2–1^	>64	>32	>32	>64	>64	>64	>64	>64	>8	256	>128	>8	2	>320
W15::W61^C2–2^	>64	>32	>32	>64	>64	>64	>64	>64	>8	512	>128	>8	2	>320
W15::W61^C2–3^	>64	>32	>32	>64	>64	>64	>64	>64	>8	256	>128	>8	2	>320
*E. coli* J53	<2	4	<0.12	<8	<4	<0.12	<0.25	4	<0.12	<0.25	<4	<0.12	<0.5	<20
J53::W61^C2–1^	<2	>32	16	>64	>64	>64	>64	>64	>8	128	>128	<0.12	<0.5	<20
J53::W61^C2–2^	<2	>32	8^#^	>64	>64	>64	>64	>64	>8	128	>128	<0.12	<0.5	<20
J53::W61^C2–3^	<2	>32	8^#^	>64	>64	>64	>64	>64	>8	128	>128	>8	<0.5	>320

AMK, amikacin; AMC, Amoxicillin/clavulanate; FEP, cefepime; CSL, cefoperazone/sulbactam; FOX, cefoxitin; CAZ, ceftazidime; CRO, ceftriaxone; CXM, cefuroxime; ETP, ertapenem; IPM, imipenem; TZP, piperacillin/tazobactam; LVX, levofloxacin; TGC, tigecycline; SXT, trimethoprim/sulfamethoxazole.

*, intermediate; #, susceptible-dose dependent.

### Evolution of hybrid plasmid pW61_1 after conjugation

In the first conjugation assay, two predominant structural variants (SV-D3 and SV-D4) were identified, representing two distinct modes of hybrid plasmid rearrangement in recipient strain W15^AziR^ (Figure 6A, Supplementary Figures 5A, B, and Supplementary Table 5). SV-D3 (51,781 bp) contained a single IS*26* at its junction, a feature consistent with the formation of a translocatable unit (TU). However, the circularized D3 fragment was undetectable, suggesting that SV-D3 likely arose through IS*26*-associated replication rather than homologous recombination-mediated excision (Supplementary Figure 5C). Similarly, SV-D4 (156,185 bp) appeared to derive from IS*Ec33*-associated replication, given the absence of the circularized D4 fragment (Supplementary Figure 5C).

S1-PFGE identified four plasmids in W15::W61^C1^ and its derivatives: band 2, band 5, band 6, and a faint band 12 (lanes 14–16 in Figures 7D–F). ONT assembly identified band 6 as SV-D3 and band 2 as pW15_2. Notably, band 12 (228 kbp) likely represented an uncharacterized recombinant plasmid rather than the native pW15_1 (band 1; 203,792 bp), because pW15_1 was no longer detectable in these samples. S1-PFGE also successfully detected the D5-excised SV-D4, represented by band 5 (lanes 14–16, Figures 7D–F). Unexpectedly, the D5 region (a 9,590-bp segment: IS*26*-IS*Kpn6*-*bla*_KPC–2_-IS*Kpn27*-△*tnpR*-IS*26*-△IS*Ecp1*-*bla*_CTX–M–65_-IS*903B*-△*iroN*) was consistently absent in SV-D4, which may be related to the remodeling of sequence adjacent to the *bla*_KPC–2_ cassette. This absence would preclude binding of primer 1R, yielding no amplification in the single-copy *bla*_KPC–2_ cassette assay (Supplementary Figure 1B). However, clear bands corresponding to the two-copy *bla*_KPC–2_ cassette were detected in W15::W61^C1.1–HD10^ and W15::W61^C1.1–FD10^, suggesting the emergence of circularized TU of the single-copy *bla*_KPC–2_ cassette (the 2F/R primers can amplify circularized single-copy cassette) (Supplementary Figure 1B).

PCR targeting the prototype pW61_1 produced strong bands in W61^P1^ and its derivatives but only weak amplification in high-imipenem overnight cultures of W15::W61^C1^, indicating that pW61_1 had rearranged after transfer (Supplementary Figure 5B). Read mapping confirmed the non-complementary structures of SV-D3 and SV-D4 (peaks ranged from 24,135 to 27,236) and the absence of circularized D3 and D4 forms (gaps spanning coordinates 189,911 to 199,740) (Figure 6B). The results also supported the existence of circularized TU carrying a single-copy *bla*_KPC–2_ cassette, as no reads mapped across the two-copy *bla*_KPC–2_ cassette region of the reference (coordinates 27,236–36,727). Together, these findings are consistent with an IS*26*-mediated model wherein IS*26* is activated following the transfer of pW61_1, and mediates plasmid rearrangement through a replication mechanism (Harmer and Hall, 2024).

A second conjugation assay was performed to determine whether the hybrid plasmid rearrangement observed in the first assay was reproducible rather than incidental. When W15^AziR^ was used again as the recipient, PCR identification targeting SV-D3, SV-D4, and the prototype plasmid showed consistent results between W15::W61^C1^ and W15::W61^C2–1^ (Supplementary Figures 6A, B). The band pattern of W15::W61^C2–1^ in lane 5 was highly similar to those in lanes 14–16 by S1-PFGE (Figures 7A, D). In contrast, the remaining two transconjugants (W15::W61^C2–2^ and W15::W61^C2–3^) and their passaged derivatives exhibited divergent SV-D4-F/R amplification profiles (Supplementary Figures 6A, B). S1-PFGE showed that W15::W61^C2–2^ contained three additional plasmids migrating above band 5, in addition to band 2 and band 6 (lane 6, Figure 7A). PCR amplification and ONT assembly identified a D2-excised circularized D3 variant (167,759 bp), corresponding to band 8 (Figures 7A, C, Supplementary Figure 6C, and Supplementary Table 6). This variant also permitted the amplification of SV-D4-F/R (12,987 bp) on circularized D3, explaining the enlarged PCR bands in W15::W61^C2–2^ and its derivatives (Supplementary Figures 6A, B). Interestingly, native plasmid pW15_1 (band 1) persisted in W15::W61^C2–2^ and its derivatives but was apparently eliminated in the other transconjugants and their corresponding derivatives, likely owing to plasmid incompatibility. For W15::W61^C2–3^, it lacked band 2 and band 5 but contained an additional band 9. Sequencing revealed that band 9 was formed through IS*26*-mediated targeted conservative-cointegration or homologous recombination between plasmid band 10 (SV-D6) and plasmid band 2 (pW15_2) (Supplementary Figure 7). After serial passage, W15::W61^C2–1/2–HD10^ and W15::W61^C2–1/2–FD10^ maintained the S1-PFGE profiles of their parental strains (Figure 7A). However, W15::W61^C2–3–FD10^ resolved band 9 into band 2 and band 10.

For the recipient *E. coli* J53, its transconjugants maintained plasmid structural stability after serial passage without antibiotics but exhibited structural changes after serial passage under high-imipenem conditions (Figures 7D–F and Supplementary Table 6): in J53::W61^C2–1^, band 13 (SV-D7) expanded to band 15 (SV-I1, I1-inserted into SV-D7); in J53::W61^C2–2^, band 14 was identified as D2-excised SV-D8 and generated band 16 (SV-I2, IS*421*-inserted into SV-D8) and band 17 (I2-inserted into SV-D9); in J53::W61^C2–3^, band 6 generated band 18 (SV-D9). In summary, in the second conjugation assay, one of the six transconjugants recapitulated the evolutionary trajectory observed in W15::W61^C1^, whereas the remaining five displayed distinct pW61_1 disintegration pathways. This heterogeneity suggests that the rearrangement outcomes are stochastic rather than strictly recipient-specific. The genetic features of the S1-PFGE profiles are summarized in Supplementary Table 6.

## Discussion

In Gram-negative bacteria, conjugative ssDNA transfer relies on three donor-cell complexes encoded by the *tra* region: relaxosome components, a membrane-embedded transport machinery (type IV secretion system, T4SS), and the type IV coupling protein (T4CP) (Virolle et al., 2020). [44] Phylogenetically, Proteobacterial T4SSs fall into eight monophyletic groups based on the VirB4 ATPase (Guglielmini et al., 2013). Among these, F-like systems assemble long, flexible pili that mediate efficient transfer in liquid culture, whereas *Agrobacterium tumefaciens* Ti-like systems produce short, rigid pili that favor transfer on solid surfaces (Virolle et al., 2020). Notably, pW61_1 and its host encode representatives of both systems. In W61, pW61_2 retains an intact F-like transfer gene cluster (Figure 5B), and the ancestral module 2 also contains a complete F-like *tra* region although its *traI* gene is now truncated by the integration of module 1 in pW61_1. Sequence analysis revealed substantial genetic divergence between the two *tra* gene clusters, suggesting distinct lineage origins. Interestingly, we found another conjugative system (with a sequence similar to pMK101-IncN) in module 3, which produces a Ti-like pilus (Vadakkepat et al., 2024; Garcillán-Barcia et al., 2025). This type of ssDNA conjugative system is powered by three cytoplasmic ATPases: VirB4, VirD4, and/or VirB11. Among them, VirD4 acts as a T4CP and recruits substrates to the T4SS channel (Redzej et al., 2017; Macé et al., 2022). In module 3, however, we characterized *virB1*-*virB11* but not *virD4*. Homology analysis revealed that the W61 chromosome (positions 1,907,318–1,919,451) harbored an inactivated Ti-like T4SS organized as *virD4*-*trbM*-*cag12*-*virB11*-*virB10*-*virB9*-*virB8*-△*virB6*-IS*Kpn26*-△*virB3*-*virB2*-*virB1*, a structure also present in the chromosomes of W15 and *K. pneumoniae* subsp. *Pneumoniae* HS11286. It remains unclear whether these chromosomal genes are functional, in particular whether the chromosomal VirD4 can couple with a plasmid-encoded relaxase. In addition, the respective and potential synergistic contributions of the two F-like T4SSs in the horizontal transfer of pW61_1 still need further investigation.

In host cells, newly synthesized dsDNA is targeted and methylated by methyltransferases of the restriction-modification (RM) system (Johnston et al., 2019; Shaw et al., 2023). Methylation appears to further restrict the activation of plasmid-borne transposons and insertion elements (Yin et al., 1988; Gao Q. et al., 2023). During conjugation, the transferred ssDNA (T-strand) retains its methylation state and then circularizes, and is converted into dsDNA, leading to plasmid establishment. We speculate that, during phenotypic conversion of the recipient, the newly established plasmid undergoes extensive replication that may lower the methylation level of nascent DNA strands and prolong the hemimethylated state (Roberts et al., 1985). Further work is required to determine whether IS*26*-mediated rearrangement is linked to the methylation status of the established plasmid in the recipient. The structural instability of pW61_1 appeared to be independent of replicative incompatibility between its modules and the native plasmids of recipient W15^AziR^. In W15::W61^C1^, the disintegration of pW61_1 may be explained by a conflict between the replicons in pW61_1 and pW15_1 [i.e., the conflict between IncFIB (pB171) and IncFIB(K), as well as between IncFII (pHN7A8) and IncFII (K)] (Supplementary Figure 2 and Supplementary Table 2). However, in W15::W61^C2–2^ and its serially passaged derivatives, S1-PFGE analysis revealed that band 6 corresponding to the plasmid carrying IncFII (pHN7A8) and IncN2, band 8 to the plasmid carrying IncFIB (pB171), and band 1 to the plasmid pW15_1, suggesting that plasmids previously thought to be incompatible can actually coexist (lane 6, 9, and 12, Figure 7A). This observation indicates not only competition between plasmids carrying the two replicon types, but also factors beyond competition contribute to their coexistence. The coexistence outcome may be explained by SV-D3 initiating replication primarily at IncN2, while pW15_1 switches to IncFII(K). However, more experimental evidence is needed to clarify the possibility of this replicon switching. Moreover, pW61_1 also rearranged in plasmid-free *E. coli* J53, indicating that the disintegration observed here was not driven by plasmid incompatibility.

Our data suggested that IS*26* played an important role in both the assembly and the rearrangement of pW61_1. Although we did not directly observe IS*26* activation, copy-in cointegration, or replication-associated excision, the conserved 8-bp direct repeat sequences at the inter-module junctions of pW61_1 were consistent with IS*26-*mediated copy-in cointegration model. Moreover, the non-complementary structures of SV-D3 and SV-D4, along with the absence of detectable circularized D3 and D4 forms strongly suggested that this plasmid rearranged via a replication-associated excision model.

**FIGURE 8:**
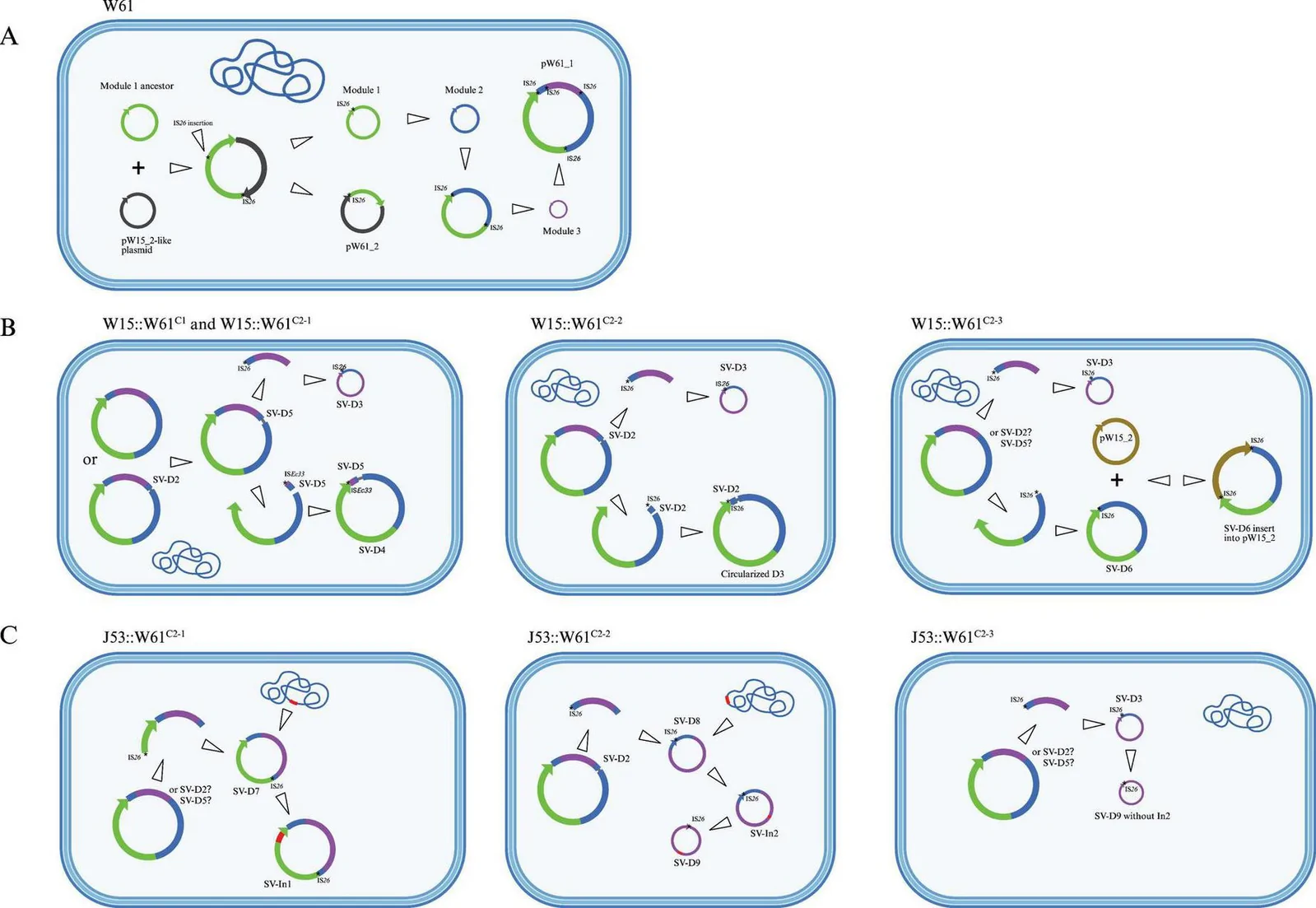
Proposed origin and disintegration of pW61_1. **(A)** Evolutionary history of pW61_1. **(B)** Schematic diagram of pW61_1 disintegrations in W15^AziR^. **(C)** Schematic overview of pW61_1 disintegrations in *E. coli* J53. Created in BioRender. Wang, P. (2026) https://BioRender.com/666uq10.

Extensive insertion of IS*26* elements into a plasmid may drive modularization of its structure. When such plasmids are transferred into hosts with a new genetic background, they tend to rapidly undergo rearrangements at different IS*26* sites, generating smaller SVs (Figures 6A, 8). Mapping IS*26* elements at the boundaries of different SV types on the pW61_1 sequence revealed that they occur in various positional combinations (seven of the 12 IS*26* elements were involved in rearrangement events, generating seven distinct SV classes; Figure 6A, top). However, certain combinations were not observed, possibly owing to the limited number of transconjugants examined here, or because such combinations had lost genes essential for replicon function or plasmid stability and therefore gradually lost their competitive advantage over successive divisions. Our findings suggest that these rearrangements are likely stochastic and subject to a founder effect.

Review of the medical records revealed that Patient 1 was transferred to the hospital from a remote village following an acute stroke. Family members reported that apart from hypertension, the patient had otherwise been in good health. However, on the second postoperative day, the patient developed a CRKP infection, and a susceptible W15 strain was isolated. Comparison of hospitalization records of patient 1 and patient 2 showed that although they were admitted to the same department, they were housed in different wards. The two clinical isolates, W61 and W15, differed by only 11 chromosomal SNPs and a few small insertions, indicating that they were clonally related. Nevertheless, owing to the absence of environment sampling, longitudinal isolate collection, and broader hospital surveillance data, the origins of the three modules of pW61_1 and the detailed assembly process of the plasmid remain speculative. Our findings highlight the importance of continuous monitoring of the nosocomial transmission of drug-resistant microorganisms in healthcare settings.

The emergence of pW61_1-like plasmids could represent an emerging threat to global public health. The co-localization of two high-risk carbapenemase genes in W61 may facilitate the transmission of carbapenem resistance. Under experimental conditions, W61 exhibited a high carbapenem-resistance retention rate and remarkable structural stability of its hybrid plasmid pW61_1, suggesting that it may retain these properties during colonization in healthcare settings and could serve as a reservoir for carbapenemase genes. In our laboratory transfer experiments, the transferred hybrid plasmid rapidly formed smaller SVs adapted to the new hosts. Whether this transfer and rearrangement also occur within patients or on surfaces in healthcare facilities remains to be investigated.

## Conclusion

In this study, we identified a novel IS*26*-assembled fusion plasmid, carrying both *bla*_NDM–1_ and *bla*_KPC–2_, from a clinically, highly carbapenem-resistant *K. pneumoniae* strain. Under laboratory conditions, this plasmid could be transferred and subsequently rearranged into smaller, resistance-retaining SVs underscoring the remarkable adaptability of such plasmids. These findings highlight a potential evolutionary trend among carbapenem-resistance plasmids and underscore the need for further research on hybrid-plasmid-mediated dissemination of carbapenem-resistance genes.

## Data Availability

The datasets presented in this study can be found in online repositories; within the NCBI with accession numbers SRS30164463, SRS30164464, SRS30112195, SRS30112192, SRS30112193, SRS30112194, SRS30112196, SRS30112191.

## Footnotes

1
https://bigsdb.pasteur.fr/

2
http://www.mgc.ac.cn/VFs/

3
https://card.mcmaster.ca/

4
https://ftp.ncbi.nlm.nih.gov/genomes/refseq/plasmid/

5
https://proksee.ca/

6
https://tncentral.ncc.unesp.br/ISfinder/howto.php

7
https://blast.ncbi.nlm.nih.gov/Blast.cgi

## Supplementary material

Supplementary Figure 1 PCR analysis of single-copy and two-copy *bla*_KPC–2_ cassette configurations and schematic diagram of primer-binding sites. **(A**) Primer-binding sites for distinguishing single-copy and two-copy configurations of the *bla*_KPC–2_ gene cassette. **(B)** Genotyping results for single-copy and two-copy *bla*_KPC–2_ cassette configurations among different strains. 1F/1R generated positive bands of 6,707 bp in templates with a single-copy *bla*_KPC–2_ gene cassette(two-copy configuration yields 11,453 bp), whereas 2F/2R produced strong 2,666 bp bands only in W61^P0^ and W61^P0.3–HD10^.

Supplementary Figure 2 Sequence homology comparison between strains W15 and W61.

Supplementary Figure 3 Carbapenem-resistance retention rate of strains W61, KP21, and KP82. # indicates *p* < 0.001 for the difference in carbapenem resistance retention rate between strain W61 and KP21, and ^ indicates *p* < 0.01 for the difference in carbapenem resistance retention rate between strain W61 and KP82.

Supplementary Figure 4 ONT read mapping for DS-W61^P0^ and DS-W61^P0.1/2/3–HD10^.

Supplementary Figure 5 PCR analysis of the presence of SV-D3, SV-D4, and the prototype pW61_1, and verification of the presence of circularized D3 and D4 in the first-round transconjugant and its derivatives. **(A)** Detection of SV-D3, SV-D4, and the prototype pW61_1 in W61^P1^ and its serially passaged derivatives. **(B)** Detection of SV-D3, SV-D4, and the prototype pW61_1 in W15::W61^C1^ and its serially passaged derivatives. **(C)** Detection of circularized D3 (CY-D3-F/R) and circularized D4 (CY-D4-F/R) in W15::W61^C1^ and its serially passaged derivatives.

Supplementary Figure 6 PCR analysis of SV-D3, SV-D4, and the prototype pW61_1 in the second-round transconjugants and their derivatives. **(A)** Detection of SV-D4, SV-D3, and the prototype pW61_1 in W15::W61^C2^. **(B**) Detection of SV-D4, SV-D3, and the prototype pW61_1 in the serial passage derivatives of W15::W61^C2^. **(C)** Detection of circularized D3 (CY-D3-F/R) and circularized D4 (CY-D4-F/R) in W15::W61^C2^ and its serial passage derivatives.

Supplementary Figure 7 Schematic diagram of the genetic structure of plasmid band 9.
