# Supplementary material for: An IS26-assembled three-module chimeric plasmid co-localizing blaNDM–1 and blaKPC–2 undergoes post-transfer rearrangement in Klebsiella pneumoniae
Source: Front Microbiol. 2026 Sep 10;17:1916718. doi: 10.3389/fmicb.2026.1916718 (PMC13601294; doi:10.3389/fmicb.2026.1916718)
Supplement: Supplementary file 1 [file Data_Sheet_1.pdf]

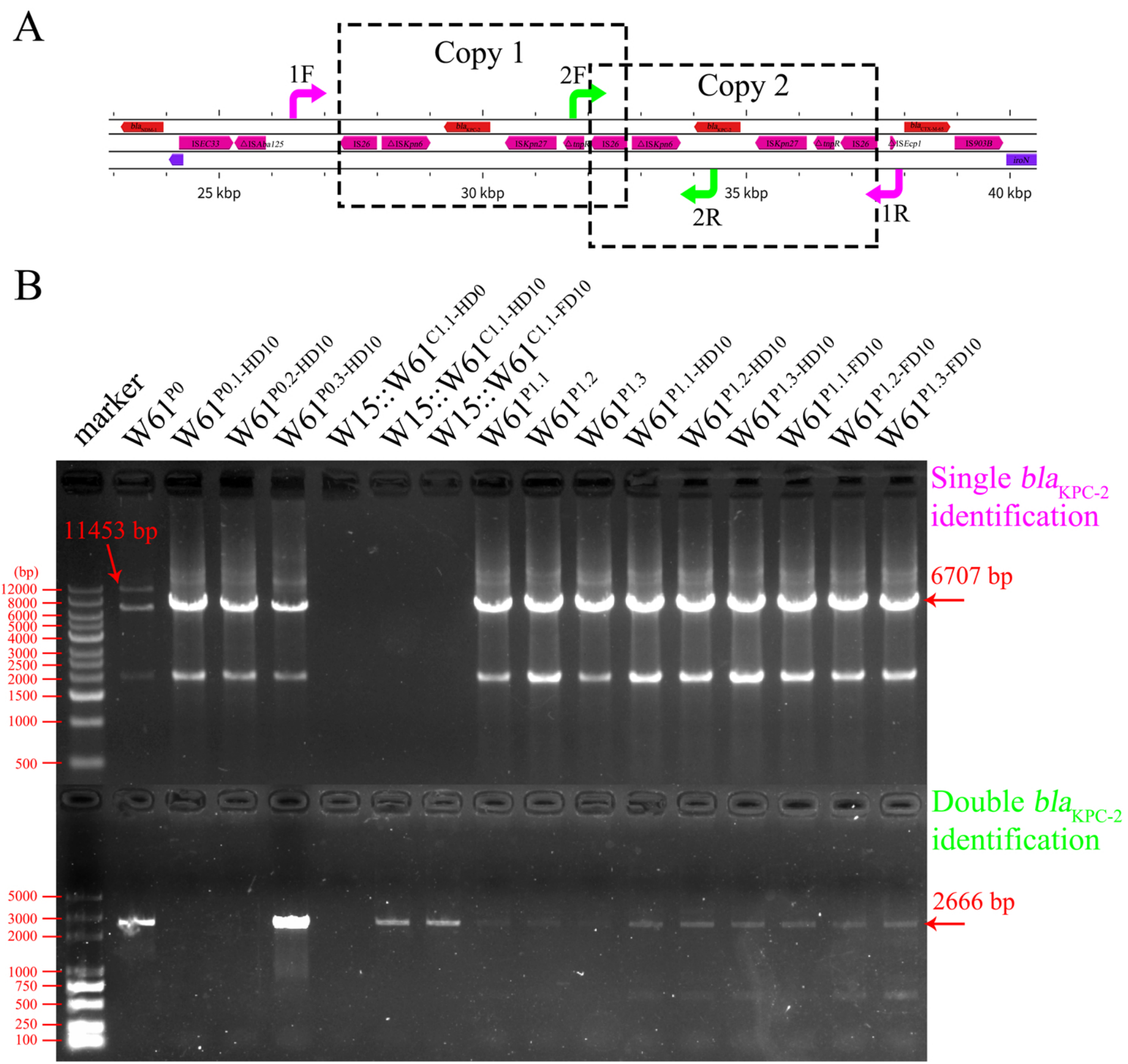
A
Copy 1
Copy 2
1F
2F
2R
1R
ISEC33
ΔISAba125
IS26
ΔISKpn6
ISKpn27
ΔtnpR
ΔISEcp1
IS903B
iroN
25 kbp
30 kbp
35 kbp
40 kbp
B
marker
W61P0
W61P0.1-HD10
W61P0.2-HD10
W61P0.3-HD10
W15::W61C1.1-HD0
W15::W61C1.1-HD10
W15::W61C1.1-FD10
W61P1.1
W61P1.2
W61P1.3
W61P1.1-HD10
W61P1.2-HD10
W61P1.3-HD10
W61P1.1-FD10
W61P1.2-FD10
W61P1.3-FD10
11453 bp
(bp)
12000
8000
6000
5000
4000
3000
2500
2000
1500
1000
500
Single blaKPC-2 identification
6707 bp
5000
3000
2000
1000
750
500
250
100
Double blaKPC-2 identification
2666 bp
