# Supplementary material for: An IS26-assembled three-module chimeric plasmid co-localizing blaNDM–1 and blaKPC–2 undergoes post-transfer rearrangement in Klebsiella pneumoniae
Source: Front Microbiol. 2026 Sep 10;17:1916718. doi: 10.3389/fmicb.2026.1916718 (PMC13601294; doi:10.3389/fmicb.2026.1916718)
Supplement: Supplementary file 2 [file Data_Sheet_2.pdf]

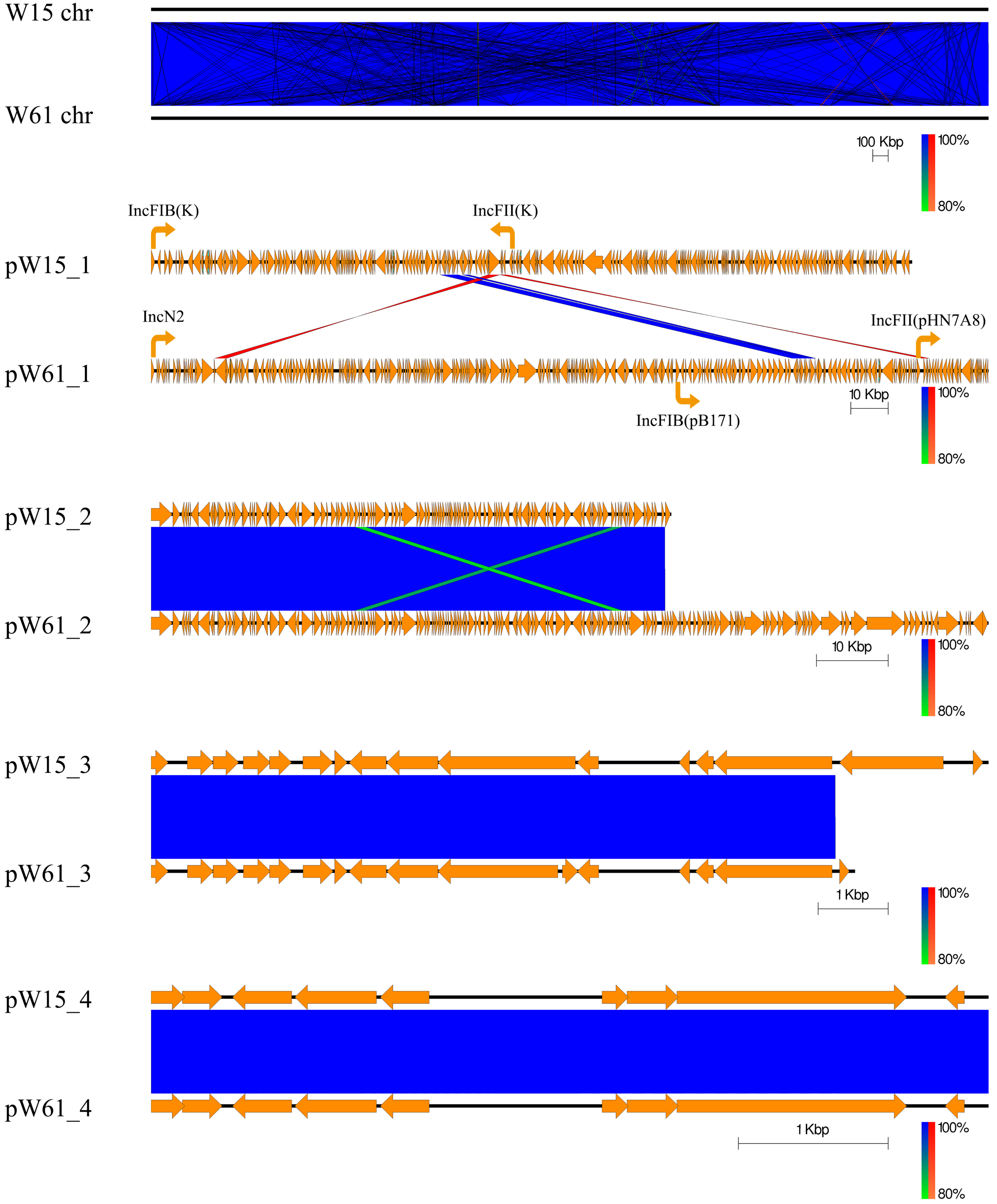
W15 chr
W61 chr
100 Kbp
100%
80%
IncFIB(K)
IncFII(K)
pW15_1
IncN2
IncFII(pHN7A8)
pW61_1
10 Kbp
IncFIB(pB171)
100%
80%
pW15_2
pW61_2
10 Kbp
100%
80%
pW15_3
pW61_3
1 Kbp
100%
80%
pW15_4
pW61_4
1 Kbp
100%
80%
