# Supplementary material for: An IS26-assembled three-module chimeric plasmid co-localizing blaNDM–1 and blaKPC–2 undergoes post-transfer rearrangement in Klebsiella pneumoniae
Source: Front Microbiol. 2026 Sep 10;17:1916718. doi: 10.3389/fmicb.2026.1916718 (PMC13601294; doi:10.3389/fmicb.2026.1916718)
Supplement: Supplementary file 3 [file Data_Sheet_3.pdf]

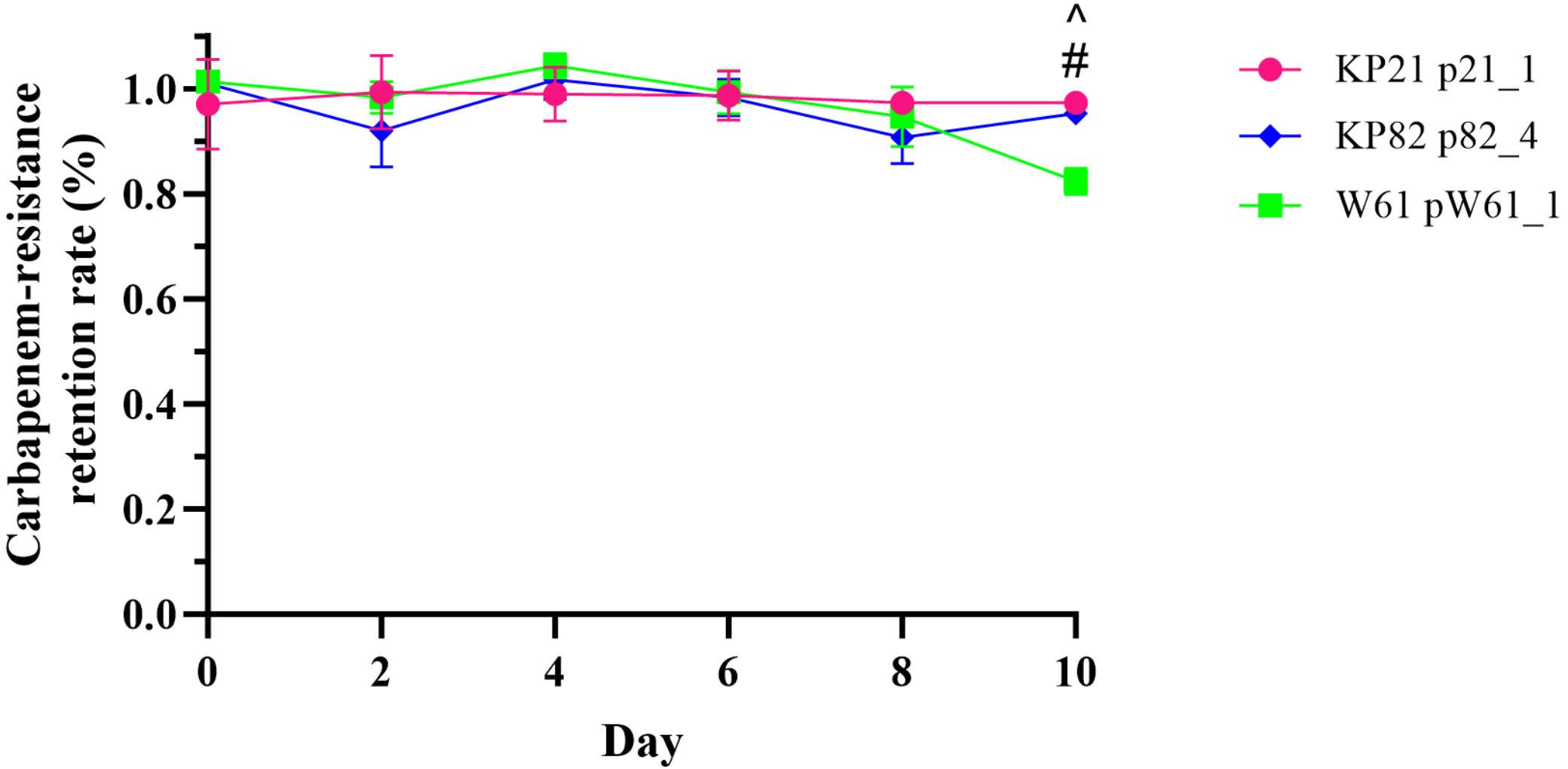
Carbapenem-resistance retention rate (%)
1.0
0.8
0.6
0.4
0.2
0.0
0
2
4
6
8
10
Day
^
#
KP21 p21_1
KP82 p82_4
W61 pW61_1
