# Supplementary material for: An IS26-assembled three-module chimeric plasmid co-localizing blaNDM–1 and blaKPC–2 undergoes post-transfer rearrangement in Klebsiella pneumoniae
Source: Front Microbiol. 2026 Sep 10;17:1916718. doi: 10.3389/fmicb.2026.1916718 (PMC13601294; doi:10.3389/fmicb.2026.1916718)
Supplement: Supplementary file 4 [file Data_Sheet_4.pdf]

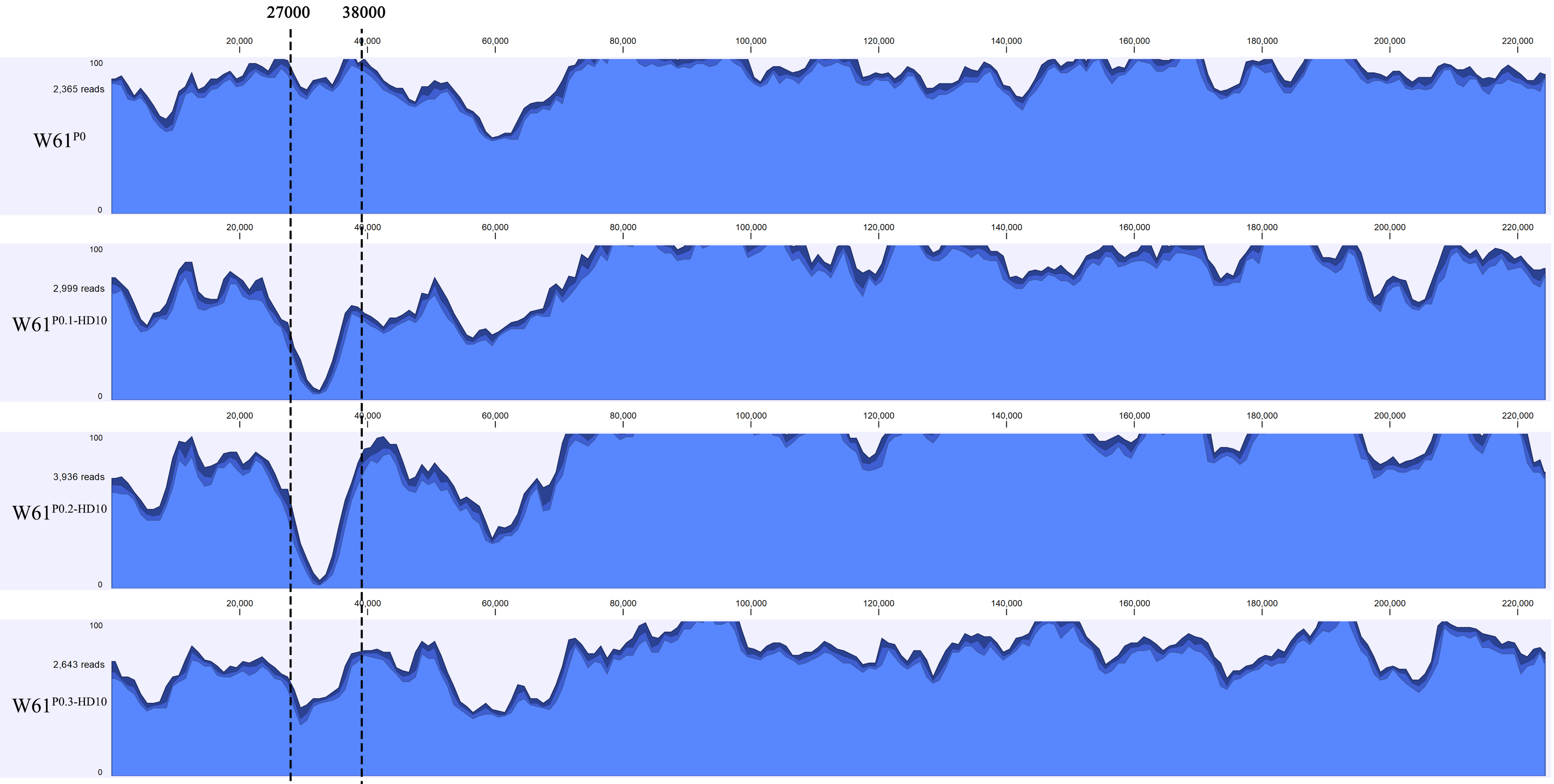
27000
38000
W61P0
2,365 reads
W61P0.1-HD10
2,999 reads
W61P0.2-HD10
3,936 reads
W61P0.3-HD10
2,643 reads
100
0
20,000
40,000
60,000
80,000
100,000
120,000
140,000
160,000
180,000
200,000
220,000
