# Supplementary material for: An IS26-assembled three-module chimeric plasmid co-localizing blaNDM–1 and blaKPC–2 undergoes post-transfer rearrangement in Klebsiella pneumoniae
Source: Front Microbiol. 2026 Sep 10;17:1916718. doi: 10.3389/fmicb.2026.1916718 (PMC13601294; doi:10.3389/fmicb.2026.1916718)
Supplement: Supplementary file 5 [file Data_Sheet_5.pdf]

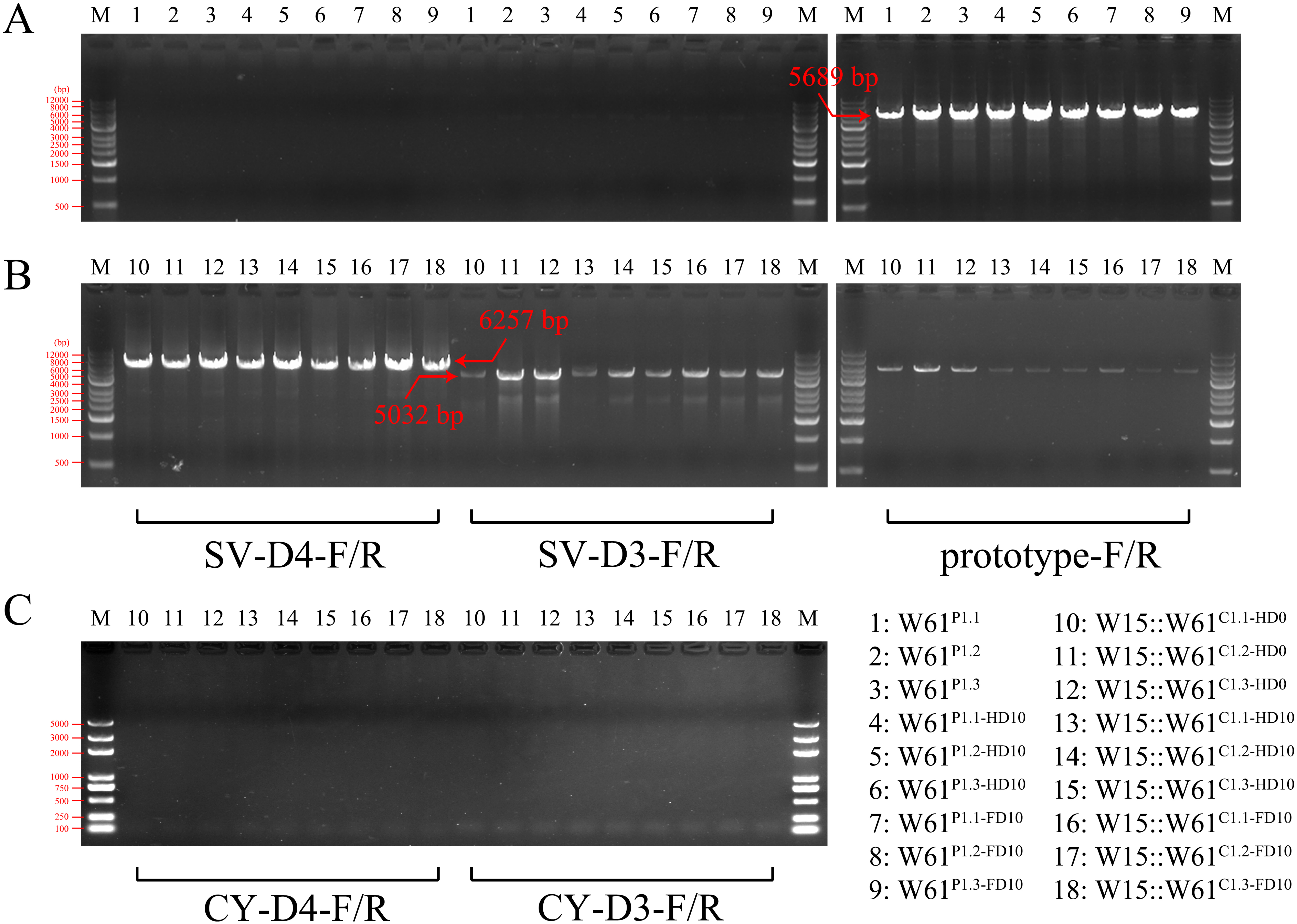
A
B
C
M
(bp)
12000
8000
6000
5000
4000
3000
2500
2000
1500
1000
500
750
250
100
5689 bp
6257 bp
5032 bp
SV-D4-F/R
SV-D3-F/R
prototype-F/R
CY-D4-F/R
CY-D3-F/R
1: $W61^{P1.1}$
2: $W61^{P1.2}$
3: $W61^{P1.3}$
4: $W61^{P1.1-HD10}$
5: $W61^{P1.2-HD10}$
6: $W61^{P1.3-HD10}$
7: $W61^{P1.1-FD10}$
8: $W61^{P1.2-FD10}$
9: $W61^{P1.3-FD10}$
10: $W15::W61^{C1.1-HD0}$
11: $W15::W61^{C1.2-HD0}$
12: $W15::W61^{C1.3-HD0}$
13: $W15::W61^{C1.1-HD10}$
14: $W15::W61^{C1.2-HD10}$
15: $W15::W61^{C1.3-HD10}$
16: $W15::W61^{C1.1-FD10}$
17: $W15::W61^{C1.2-FD10}$
18: $W15::W61^{C1.3-FD10}$
