# Supplementary material for: An IS26-assembled three-module chimeric plasmid co-localizing blaNDM–1 and blaKPC–2 undergoes post-transfer rearrangement in Klebsiella pneumoniae
Source: Front Microbiol. 2026 Sep 10;17:1916718. doi: 10.3389/fmicb.2026.1916718 (PMC13601294; doi:10.3389/fmicb.2026.1916718)
Supplement: Supplementary file 6 [file Data_Sheet_6.pdf]

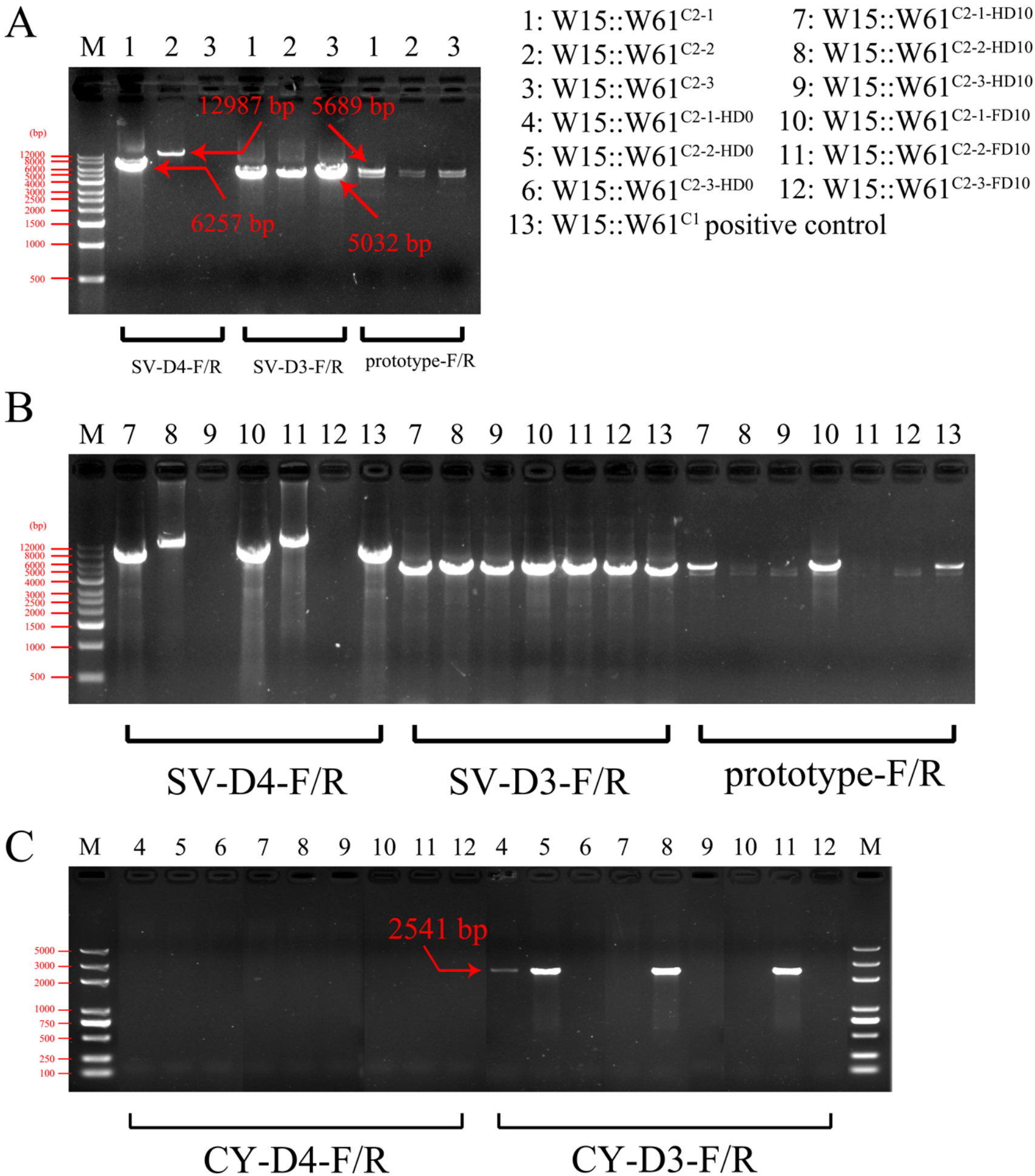

A
M 1 2 3 1 2 3 1 2 3
(bp)
12000
8000
6000
5000
4000
3000
2500
2000
1500
1000
500
12987 bp
5689 bp
6257 bp
5032 bp
SV-D4-F/R
SV-D3-F/R
prototype-F/R
1: W15::W61C2-1
2: W15::W61C2-2
3: W15::W61C2-3
4: W15::W61C2-1-HD0
5: W15::W61C2-2-HD0
6: W15::W61C2-3-HD0
7: W15::W61C2-1-HD10
8: W15::W61C2-2-HD10
9: W15::W61C2-3-HD10
10: W15::W61C2-1-FD10
11: W15::W61C2-2-FD10
12: W15::W61C2-3-FD10
13: W15::W61C1 positive control
B
M 7 8 9 10 11 12 13 7 8 9 10 11 12 13 7 8 9 10 11 12 13
(bp)
12000
8000
6000
5000
4000
3000
2500
2000
1500
1000
500
SV-D4-F/R
SV-D3-F/R
prototype-F/R
C
M 4 5 6 7 8 9 10 11 12 4 5 6 7 8 9 10 11 12 M
5000
3000
2000
1000
750
500
250
100
2541 bp
CY-D4-F/R
CY-D3-F/R
