# Supplementary material for: An IS26-assembled three-module chimeric plasmid co-localizing blaNDM–1 and blaKPC–2 undergoes post-transfer rearrangement in Klebsiella pneumoniae
Source: Front Microbiol. 2026 Sep 10;17:1916718. doi: 10.3389/fmicb.2026.1916718 (PMC13601294; doi:10.3389/fmicb.2026.1916718)

pW15_2 boundary: --------TGAGCAAC-IRL-IS*26*-IRR-AAGCTGGC--------

SV-D6 boundary: --------TGAGCAAC-IRL-IS*26*-IRR-AATTACAG--------

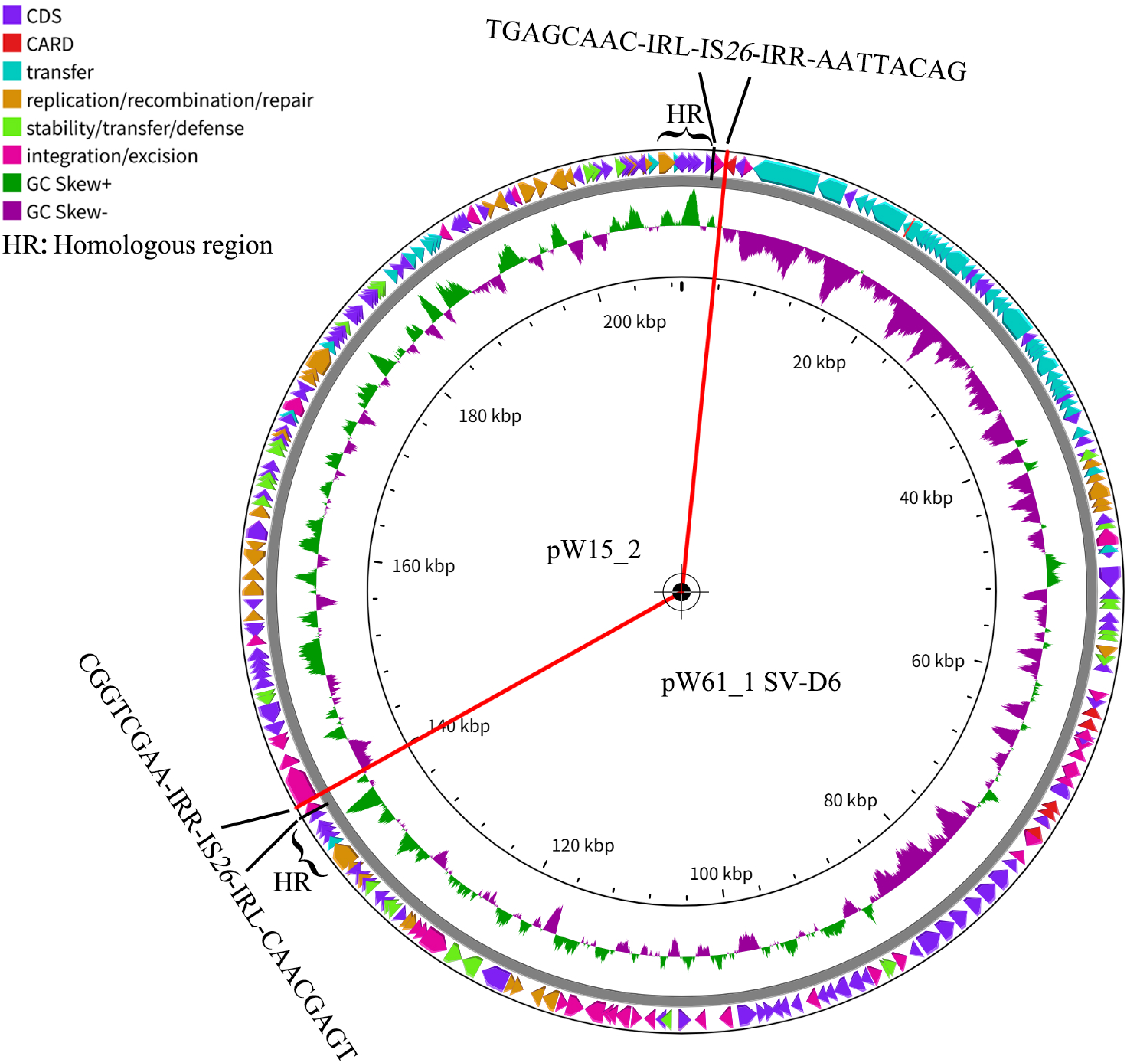

Supplement: Supplementary file 7 [file Data_Sheet_7.pdf]
